# Optimal maximum power point tracking strategy based on greater cane rat algorithm for wind energy conversion system

**DOI:** 10.1038/s41598-025-18710-7

**Published:** 2025-09-12

**Authors:** Kareem M. AboRas, Mohammed Hassan EL-Banna, Ashraf Ibrahim Megahed, Muhammad R. Hammad

**Affiliations:** Electrical Power and Machines Department, Faculty of Engineering, Alexandria University, Alexandria, 21544 Egypt

**Keywords:** Wind energy systems, GCRA, MPPT, Optimization techniques, PMSG, Electrical and electronic engineering, Energy grids and networks

## Abstract

With the rapidly increasing usage of renewable sources, especially wind power, maximizing the power produced from wind energy conversion system (WECS) has become a major concern. Various methods are utilized in the domain of wind turbine performance enhancement for tracking the maximum power point (MPP). Among them, the perturb and observe (P&O) approach is widely applied because of its straightforward implementation. Nevertheless, the primary drawback of this approach is the imprecision caused by variations at the peak power point. Consequently, due to wind’s arbitrary and complicated characteristics, using an intelligent optimization technique is compulsory as it can give effective tracking performance. In this study, a recently developed nature-inspired metaheuristic, termed the Greater Cane Rat Algorithm (GCRA), which emulates the cognitive foraging behavior of greater cane rats during and after the breeding season. The GCRA approach seeks to regulate the boost converter by computing the duty cycle value using the voltage and current variables. The Wind Energy Conversion System (WECS) incorporates a wind turbine, a Permanent Magnet Synchronous Generator (PMSG), a rectifier, and a DC/DC boost converter that is linked to a load. The wind system can track the maximum power via a mechanical sensorless tracker system without the need to connect an additional mechanical sensor. The suggested strategy is compared to various tracking methodologies, including the classical Perturb & Observe (P&O), Particle Swarm Optimization (PSO), and Gray Wolf Optimization (GWO). The obtained results, which have been executed in the environment of MATLAB/SIMULINK R2022b, illustrate that the proposed approach improves the performance of the tracking system under different wind profiles step, realistic, and ramp variation of the wind velocity. The proposed strategy outperforms a tracking efficiency that exceeds 99%, surpassing other considered tracking approaches, which are at 95.5%, 94.7%, and 91.4% with the least error ratio and the best tracking for the power coefficient ratio.

## Introduction

### Background and literature review

The daily continuing increase in world population has a significant influence on worldwide electrical energy consumption^1^. The utilization of electrical energy is an indicator of a country’s technical advancement^2^. Global energy consumption is continuously increasing as a result of technical breakthroughs and increased industrial production in civilizations. Although traditional fossil fuel-based power generation has historically dominated energy systems^3^,^4^, this tendency has begun to shift in recent years. Traditional sources of energy are depleting and are inadequate to fulfill expanding energy demand, and their usefulness is decreasing. However, the usage of these sources raises level of carbon dioxide emission and contributes to significant anxieties on surrounding environment for instance the current global warming^2^. At this time, renewable sources of energy (RESs) have a crucial role, and hence their usage throughout the world has a great expanding^5^–^8^. Among the many RES technologies, wind energy is receiving greater attention since it is a clean, infinite energy source with approximately no emission of carbon and requires fewer resources such as the land. As a result, the effective utilization of wind energy to meet the demand increasing of energy necessitates more study in this field.

Wind energy conversion system (WECS) is a scheme designed to achieve efficient conversion of aerodynamic energy from airflow into a different form of energy, such as electrical energy. However, wind velocity has intrinsically unpredictable dynamics, making problematic for the control of WECS. In terms of structure, numerous topologies of WECS are stated in the literature. These topologies are categorized into constant velocity WECS and variable velocity WECS^9^. Although the constant-velocity type is simpler and less expensive, variable one achieves superior efficiency by reaching maximum power point (MPP) for the entire period of operation duration at all values of speed.

There are several instances of variable-velocity WECS designs that use Squirrel Cage Induction Generators (SCIG)^10^, Doubly-Fed Induction Generators (DFIG)^11^, and Permanent Magnet Synchronous Generators (PMSG)^9^,^10^,^12^. Permanent magnet types are popular globally and are especially chosen for usage in medium and small -sized schemes because their operation depends on the direct drive, competence, dependability, high density of energy, and minimal repairing requirements^9^,^13^. Because of these benefits, PMSG was used in this investigation. In studies of PMSG-based Wind Energy System, researchers have shown a preference for two primary configurations: the rectifier coupled with a DC-DC converter and the scheme of back-to-back converter (BTBC)^14^. Nevertheless, when efficiency and cost are taken into account, the uncontrolled rectifier combined with a DC-DC boost converter topology is typically favored for medium and small -sized systems. Due to their tremendous effectiveness and straightforward implementation of controller algorithms on this architecture for outperforming the maximum power from the wind turbine. MPPT operating techniques for WECS are classified into two categories: direct power control techniques and indirect power control techniques^15^. The first one doesn’t require previous information, or any further sensors related to mechanical parts, but the second techniques must^10^,^16^. The most prevalent direct power control (DPC) approaches are Incremental Conductance (IC), Perturbation and Observation (P&O)^10^, and Optimum Relation Based (ORB)^17^. In contrast, indirect power controllers (IPCs) include Tip Speed Ratio (TSR), Optimal Torque Control (OTC)^18^, and Power Signal Feedback (PSF)^19^.

However, each MPPT approach has its benefits and limitations related to rate of convergency, usage of sensors and preliminary data requirements, cost of implementation, and dependability. Several tracking systems have been developed to harvest the greatest power from the wind system. Fuzzy and its adaptiveness for PI control^20^, radial basis type of neural network^21^, artificial neural network (ANN), and reinforcement learning^22^ architectures are conductively investigated for better outcomes. However, artificial intelligence approaches have certain drawbacks, including memory and prior knowledge requirements, computational burden, expert knowledge, and learning time. In contrast, nonlinear methodologies of control for instance the backstepping approach^23^ and several sliding mode control methods^12^,^14^ have been applied in WECS.

Particle swarm optimization (PSO) was discussed in^24^ and compared to fuzzy controllers and traditional P&O. Their proposed approach showed slower dynamic response under rapidly varying wind speeds. Similarly, modified Grey Wolf Optimizer (MGWO) for MPPT was introduced in wind systems^25^ with improved convergence, but the algorithm complexity increases significantly, making real-time implementation challenging. In^26^, the second order sliding mode controller (SO-SMC) was addressed to regulate a wind turbine system and outperformed the conventional sliding mode controller (C-SMC).

The Multiple Linear Regression Approach was exploited in^27^ to calculate the peak power of a sensor-less wind system, and the findings were acquired through simulation and then by apractical experimentation. In^28^, a fuzzy twisting algorithm was applied to operate a sensor-less wind turbine to drive a feeder water pumping system, and a fuzzy super twisting algorithm was used to regulate the generator speed to its ideal value and deliver the optimum direct current. A comparison of ANN and ANFIS controllers for PMSG Variable Speed WECS was conducted in^29^, and the findings confirmed the benefit of ANFIS as an MPPT controller over ANN owing to higher extracted power, lower error, and lower total harmonic distortion. In^30^, three different topologies of continuous output buck converters were compared together, two of them were sensor-based and the third one was sensorless, and the obtained outcomes proved the efficiency of the sensorless type over the others. In^31^ investigated the use of both PSO and GA to optimize fuzzy logic control membership functions for creating MPPT in a wind energy conversion system (WECS). Grey wolf optimization (GWO)^32^ is applied to develop an MPPT controller for maximizing power extraction from a wind generation system, and the proposed algorithm demonstrates stability and effectiveness. A modified golden section search (GSS) was introduced in^33^ as an MPPT controller for a permanent magnet synchronous generator-based WECS. It detects wind speed variations, adapts to dynamic uncertainty ranges, and minimizes oscillations around the maximum power point. The effectiveness of the proposed method is validated across various wind scenarios, including sudden changes in wind speed and stochastic wind profiles. The Archimedes optimization algorithm (AOA) is applied in^34^ for MPPT simulation in a wind energy generation system, where the AOA adjusts the converter duty cycle to maximize output power. This study examines three scenarios: fixed wind speed, variable wind speed, and real wind speed data recorded at four locations in Saudi Arabia. An approach using the grasshopper optimizer (GOA) was introduce in^35^ for MPPT simulation in a wind energy generation system in the Aljouf region of Saudi Arabia. The GOA adjusts the converter duty cycle to enhance output power. Various locations within the Aljouf region were analyzed, and the proposed method was compared with other approaches. Improved hybrid direct power control-backstepping (DPC-BS) control strategy was presented in^36^ to discuss optimization of the extraction of wind energy in real circumstances and push the doubly-fed induction generator (DFIG) to work appropriately with outperforming and high robustness.

### Main contributions

This work proposes a novel MPPT controller technique for a standalone sensor-less wind energy conversion system (WECS). This controller exploits Greater Cane Rat Algorithm (GCRA)^37^, a novel meta-heuristic approach for optimization which is inspired by animal behavior for seeking food and foraging. Under the umbrella of preceding information, the following is a collection of the main contributions that this study has made:

1. First-of-its-kind application of a recently-published nature-inspired metaheuristic optimization technique, greater cane rat algorithm (GCRA), is deployed to improve power tracking in a system of wind turbine feeding a permanent magnet synchronous generator (PMSG) without mechanical sensors.
2. The suggested method demonstrates robustness by addressing MPP in various environmental situations, including step fluctuation of wind speed and realistic variation. Additionally, it is compared with other well-known algorithms to demonstrate its superiority among these techniques in the field of maximum power tracking.
3. The manuscript is formed as follows: Following the first few lines of Section “Introduction”, Section “The studied WECS model” explicates the model used to investigate the WECS system and each component inside it. Section “Greater cane rat algorithm (CGRA)” explains how the greater cane rat algorithm (GCRA) is applied to improve the proposed MPPT control technique. Section “Results and discussion” describes obtained results, assessments, contrasts, and comparisons with other methodologies. Section "Conclusion" is the study’s last section, which includes detailed conclusions of the work done.

## The studied WECS model

**Fig. 1:**
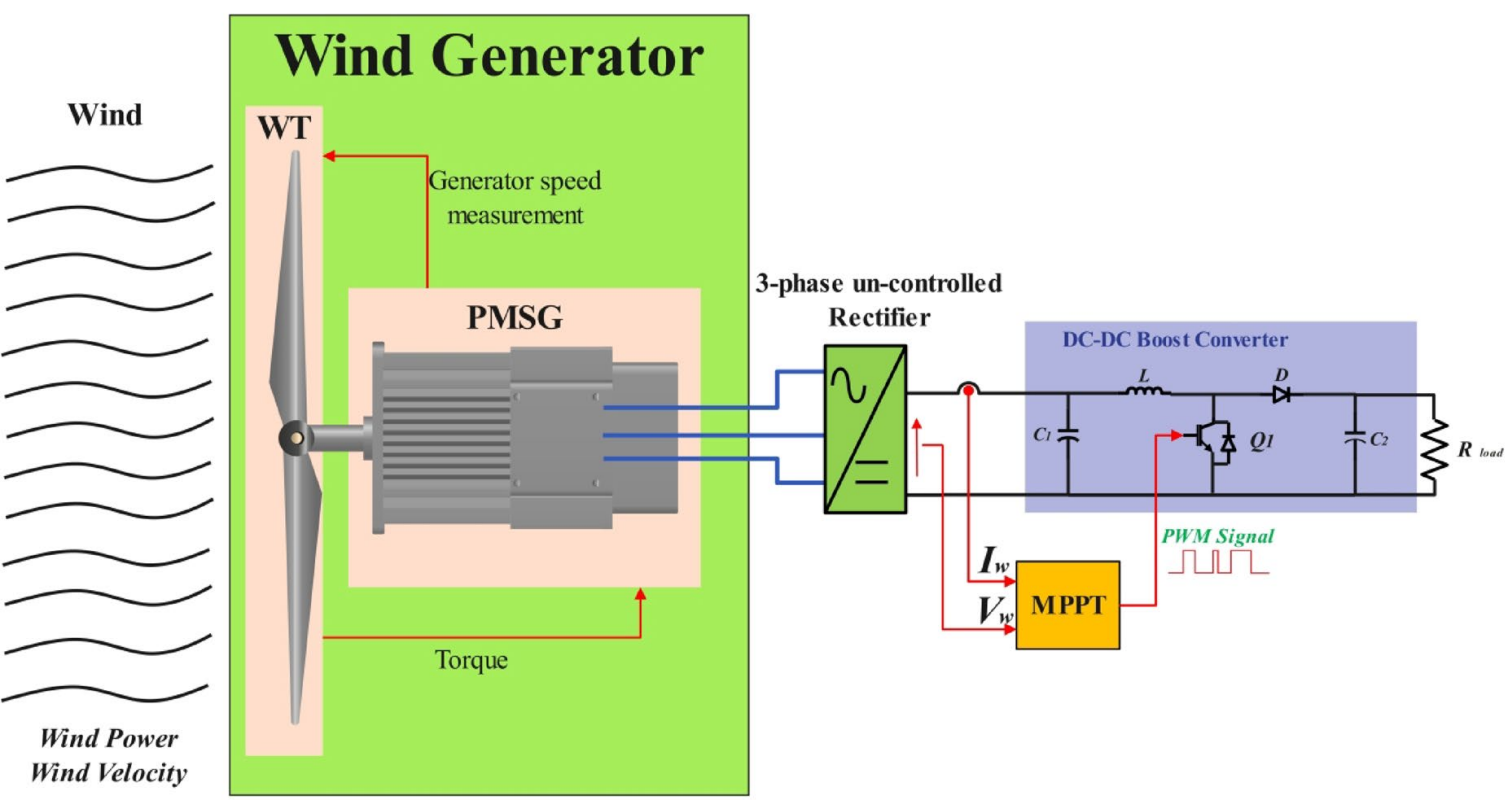
Sensorless MPPT for Wind Energy Conversion System WT: Wind Turbine. PMSG: Permanent Magnet Synchronous Generator.

This section describes the system that will be analyzed for the research. Fig. 1 displays whole system under investigation. It is a small, stand-alone wind energy conversion system (WECS) based on a permanent magnet synchronous generator (PMSG) directly derived by a small wind turbine, and the whole system is utilized to supply a linear resistive load. All system components such as the wind turbine, PMSG, uncontrolled rectifier, connecting capacitor, boost DC-DC converter, and the associated controller for MPPT are thoroughly explained in the next subsections.

### Wind turbine attributes

A wind turbine derives the mechanical power it needs from the rotational force generated by the air which hits its blades. A turbine can generate the mechanical power according to^27^ and can be expressed by Eq. (1):

1 $${P}_{m}=\frac{1}{2}\rho A{C}_{p}(\uplambda , \beta ){v}_{w}^{3}$$

**Fig. 2:**
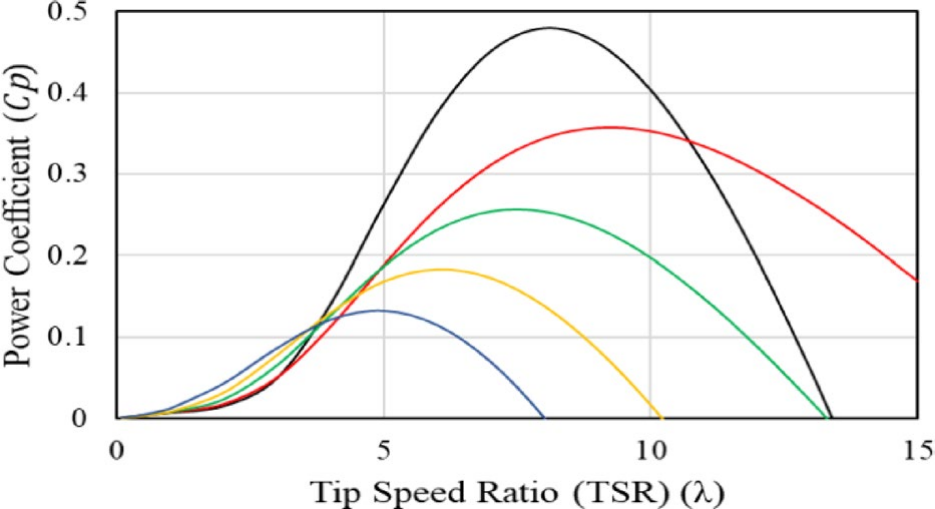
Relation between power coefficient and tip speed ratio of wind turbine at different pitch angle.

**Fig. 3:**
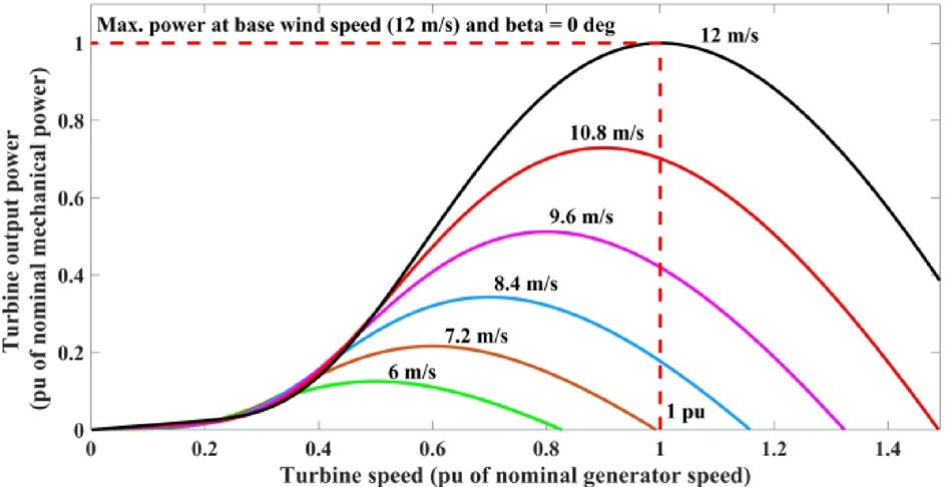
Turbine output power characteristics as a function of speed of turbine at various speeds of wind.

In this equation, ${v}_{w}$ represents wind speed, $\rho$ represents density of the air, $A$ is area of the turbine, and ${C}_{p}(\uplambda , \beta )$ expresses the coefficient of power for the turbine, includes *β* and λ which are angle of the pitch and tip speed ration (TSR), respectively. Relation between tip speed ration and power coefficient of wind turbine can be described according in^26^ which is mentioned in Eq. (2).

2 $$\left\{\begin{array}{c}{C}_{p}\left(\uplambda , \beta \right)={C}_{1}\left(\frac{{C}_{2}}{{\uplambda }_{i}}-{C}_{3} \beta -{C}_{4}\right){e}^{-\left(\frac{{C}_{5}}{{\uplambda }_{i}}\right)}+{\text{C}}_{6}\lambda \\ \frac{1}{{\uplambda }_{i}}=\frac{1}{\uplambda +0.08\beta }-\frac{0.035}{{\beta }^{3}+1}\end{array}\right.$$

where constants have the values of $${{\text{C}}_{1}= 0.5176,\text{ C}}_{2}= 116, {{\text{C}}_{3}= 0.4,\text{ C}}_{4}=5, {{\text{C}}_{5}=21,\text{ C}}_{6 }= 0.0068$$^37^. $${C}_{p}$$ changes exclusively with λ in the wind turbine. According to Eq. (2), Fig. 2 depicts the relation between the power coefficient ($${C}_{P})$$ as a function of the speed tip ratio (STR) ($$\uplambda )$$ at different values of pitch angel of the turbine ($$\beta )$$. From the figure, it is clear that in the case of fixed wind turbine (i.e., $$\beta =0$$), the maximum power coefficient equals 0.48 and this can be achieved at the speed tip ratio ($$\uplambda$$) equals 8.1 which can be expressed mathematically in Eq. (3).

3 $$\uplambda =\frac{{\omega }_{m}R}{{v}_{w}}$$

where ${\omega }_{m}$ and $R$ are the rotational speed and blade radius, respectively. Additionally, the relation between the speed of the turbine and the output mechanical power that can be obtained from it at different velocities of wind can be expressed in Fig. 3. As shown in Fig. 3, at each speed of wind, only one speed of turbine is considered the optimal speed that maximizes power output. To rewrite mechanical power, substitute (3) in (1) as follows and this produces Eq. (4):

4 $${P}_{m}=\frac{1}{2}\rho A{C}_{p}(\uplambda , \beta ){(\frac{{\omega }_{m}R}{\uplambda })}^{3}$$

Swept area is substituted in Eq. (4) by $A=\pi {R}^{2}$ to obtain Eq. (5):

5 $${P}_{m}=\frac{1}{2}\frac{\rho \pi {R}^{5}{C}_{p}}{{\uplambda }^{3}}{{\omega }_{m}}^{3}$$

Hence, if $\lambda$ is set to the optimal value, ${C}_{P}$ remains maximal, and ${P}_{m\_max}$ is achieved as mentioned in Eq. (6)^38^:

6 $${P}_{m\_max}=\frac{1}{2}\frac{\rho \pi {R}^{5}{C}_{p\_max}}{{{\uplambda }_{opt}}^{3}}{{\omega }_{m}}^{3}={K}_{p\_opt}{{\omega }_{m}}^{3}$$

where ${K}_{p\_opt}$ represents a constant. However, there is one concern that must be considered. Even if ${P}_{m\_max}$ is obtained, achieving ${P}_{L\_max}$ cannot be assured. Because the generator converts the WT’s mechanical power to voltage, which is then rectified by the three-phase rectifier and attuned to the required point of operation using boost converter. Output power is calculated based on generator efficiency (${\eta }_{gen}$) and power conversion topology (${\eta }_{conv}$)^39^.

7 $${P}_{L}={P}_{m}{\eta }_{gen}{\eta }_{conv}$$

Nevertheless, because the losses change with generator speed^38^, neither the generator nor the converter efficiencies are constant. As a result, evaluating MPPT efficiency adjacent to the load is better than that beside the mechanical portion yields further precise and effectiveness findings

### Permanent magnet synchronous generator and rectifier attributes

PMSG does not necessitate any current for excitation in its stator and functions self-excited when magnets are installed in its rotor. It transforms mechanical power into electrical power while being rotated by the turbine on the same shaft, which is very appealing for WECS. PMSG’s induced back emf changes proportionately with rotor speed and is characterized in Eq. (8):

8 $$E=KP\omega$$

where $p$ denotes the number of poles` pairs and k represents a constant. Voltage-per-phase of PMSG is depicted in Eq. (9) and stated in^9^:

9 $$V=E-I({R}_{s}+jP\omega {L}_{s})$$

where ${L}_{s}$ and ${R}_{s}$ are the stator’s phase`s inductance and resistance, respectively and $I$ is the current passes through the phase. By neglecting the losses due to diode, produced voltage of the rectifier can be depicted in Eq. (10) as stated in^34^,^35^:

10 $${V}_{dc}=\frac{3\surd 6}{\pi }V-\frac{3}{\pi }P\omega {L}_{s}{I}_{L}=\frac{3\sqrt{6}}{\pi }\left(E-I\left({R}_{s}+jP\omega {L}_{s}\right)\right)-\frac{3}{\pi }P\omega {L}_{s}{I}_{L}$$

So, ${V}_{dc}$ displays the variation with $\omega$, linearely^9^, which is epitomized in Eq. (11):

11 $${{V}_{dc}=K}_{v}\omega$$

### Boost converter characteristics

To align the voltage levels, a DC-DC boost converter must be connected to the dc bus before the load. The inclusion of it in this topology is due to the fact that it enables harmless, easier, and less complexity due to it is utilizing a single element for switching control. Adjusting the control signal for this element regulates the PMSG’s output voltage. When selecting converter components, the duty cycle $D$ is an imperative factor to consider in order to exploit power output, maximally. According to^30^, voltage and current produced by the boost converter may be determined according to Eqs. (12) and (13).

12 $${V}_{out}={V}_{in} \frac{1}{1-D}$$

13 $${I}_{out}={I}_{in}(1-D)$$

By combining Eqs. (6) and (11), ${P}_{DC}$ and ${P}_{L}$ may be determined in Eqs. (14) and (15).

14 $${P}_{DC}={\eta }_{gen}{P}_{m}={\eta }_{gen}{K}_{p\_opt}{\omega }^{3}$$

15 $${P}_{L}={\eta }_{conv}{P}_{DC}={\eta }_{conv}{\eta }_{gen}{K}_{p\_opt}\frac{{V}_{dc}^{3}}{{K}_{v}^{3}}={K}_{r\_opt}{V}_{dc}^{3}$$

**Fig. 4:**
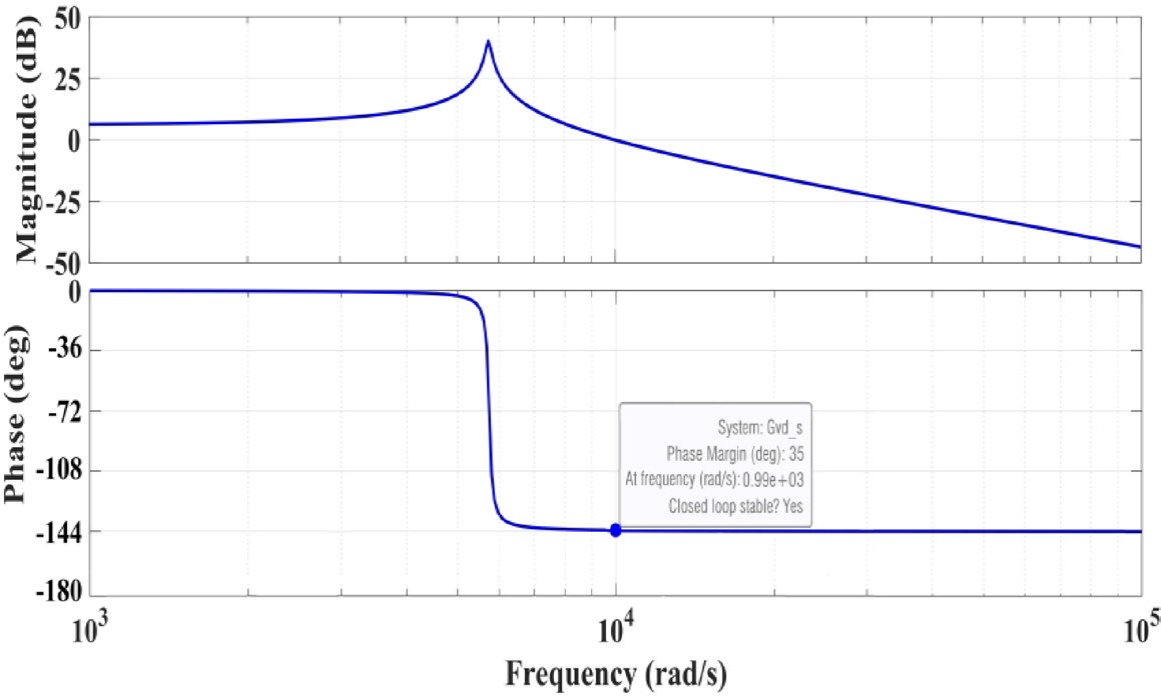
Bode plot of the boost converter in the proposed system.

As a result, tracking the ${V}_{dc\_opt}$ reference allows the system to attain its maximum power. This technique eliminates the necessity for mechanical sensors to measure variables such as wind speed. As the boost converter is an essential component in the system, it is mandatory to evaluate its stability under different operating conditions. Therefore, bode plot shown in Fig. 4 analysis the stability of it with a positive value of phase margin equal 35 that indicates a good stable condition.

## Greater cane rat algorithm (CGRA)

### Concept and inspiration

In Ghana, Nigeria, and other West African locations, the greater cane rat (CGR) is known as the grasscutter. It is a member of the cane rat or African historiography rodent family. The smaller cane rat is the second member^40^. In Sub-Saharan Africa, they dwell around beds, banks of rivers, swamplands, lakes, and long, cane has high density such as grasses^41^. The larger cane rat is one of Africa’s biggest rodents.

**Fig. 5:**
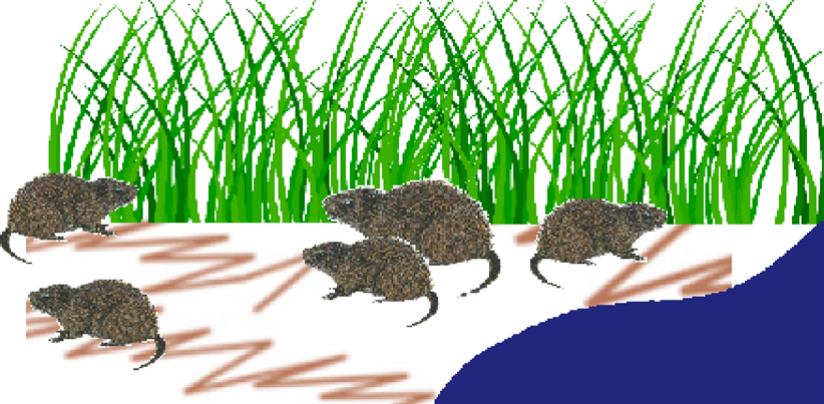
The Natural habitat of GCR.

Greater cane rats are proficient swimmers that exploit the water to avoid danger. They are very quick and nimble on land. They are generally nocturnal but may also be active throughout the whole day^42^. They are male-controlled and live in tiny families. headed by a dominating male. Rats` habitat is normally a shell in dense foliage. However, they will occasionally exploit subterranean refuges that termites or other creatures’ abandon. When they feel hazard or any danger, they groan or sprint to direction of water^43^. Bigger cane rats’ main food consists of grass, although they also consume other plants, fruits, and tree phloem. GCRs reside near a source of water, which is indicated by the darkened section at bottom of the picture shown in Fig. 5, high grasses may be noticeable, and white places and paths reflect number of methods the did to known sources of food via their characteristics.

Greater cane rats (GCR) may be regional, only males battling using their noses. Further, they are extroverted creatures, residence in assemblies that include a leading male, numerous youngsters and several females. Rats forage by cane that can be cut with particularly developed upper teeth. They are very nocturnal and have sufficient intelligence to leave paths by way of grazing among grasses and reeds. The pathways would ultimately lead to food, water sources, and housing^43^.

### Initialization of population

The GCRA optimization procedure starts with the random production of population ($X$) of Greater Cane Rats (GCR) according to Eq. (16).

16 $$X=\left[\begin{array}{ccc}{X}_{\text{1,1}} {X}_{\text{1,2}}& \cdots & {X}_{1,d}\\ \vdots & {X}_{i,j}& \vdots \\ {X}_{n,1} {X}_{n,1}& \cdots & {X}_{n,d}\end{array}\right]$$

where $X$ denotes the total population of GCR, $n$ and $d$ represent dimensions of population and problem, respectively.

Equation (17) arbitrarily generates individual (${X}_{i,j}$) in ${i }^{th}$ position with the ${j }^{th}$ length at random.

17 $${X}_{i,j}=rand*\left({UB}_{j}-{LB}_{j}\right)+{LB}_{j}$$

where $rand$ is an arbitrary value from 0 to 1, $LB$ and $UB$ are lower bound and upper bound, respectively.

### The scheme of GCRA

It displays attitude of regional, frequently participating in nose duels, which are largely between males. These organisms are sociable, establishing assemblies containing a foremost male, various youngsters, and females. They forage by wounding grasses and canes with their specially developed upper fangs. As night-time creatures, they are skilled at creating paths through grassland that lead to nutrients, and refuge. The period of exploration phase is distinguished by their emigration from numerous lodgings in the surrounding region for food searching and build routes.

Leading-male rat maintains knowledge for these tracks, to let the others adjusting places consequently. Other males are able to distinguish mating period and isolate themselves apart of their cluster. It is hypothesized that throughout this leave-taking, efforts of food searching are intense within locations with torrential supplies of food, allowing for easier exploitation. This work mathematically simulates intelligent foraging tracks and mating season behaviors in order to create the GCR methodology and accomplish optimization actions.

**Fig. 6:**
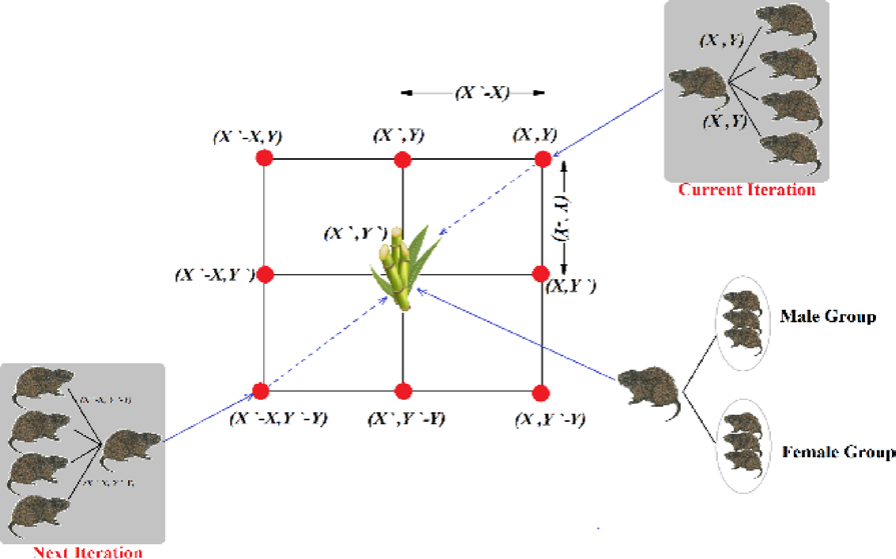
Conceptional model of GCRA.

The model of GCRA is shown in Fig. 6. The targeted source of food is supposed to be located at location ($X{\prime}, Y{\prime}$). The alpha male knows the way to this food supply, which it then passes on to the other members of the family, who change their positions according to this information. An alpha in location ($X, Y$) is awareness of the supply of nutrition at the position ($X{\prime}, Y{\prime}$).

The dominant rat is expected as fittest rat, or the vector of position gives the best optimal fitness function. This fittest leads the assembly of rats and has the previous awareness of directions of nutrition or refuge, locations of further rats are altered based on the dominant male’s position, as per Eq. (18). The position of dominating rat is represented by ${X}_{K}$. The GCRA enters either the exploration or exploitation stage based on the value of $\rho$, a variable that notifies the type of season, rainy or not. Value of $\rho$ is cautiously chosen to accomplish an equilibrium between the exploitation stage exploration stage. After conducting a thorough parametric study, the value of $\rho$ is prudently adjusted to 0.5.

18 $${X}_{i,j}^{new}=0.7 \frac{({X}_{i,j}+{X}_{K,j})}{2}$$

where ${X}_{i,j}^{new}$ indicates new location of the GCR, ${X}_{i,j}$ indicates the existing location of GCR, and ${X}_{K,j}$ denotes the leading male in the ${j}^{th}$ length.

### Exploration phase

The GCRs create refuges across their range such as riverbanks, marshlands, and planted agricultural fields. They leave the various refuges to food, either next trails to prior sources of food or searching for new sources of food and leave-taking tracks. Equation (19) shows how the position of leading male controls the new location for the remaining members of population of rats. During the rats` mobility simulation, if additional rat has a higher value of the fitness function value than the fittest member, it is mandatory to update the fittest member, and the extra rats’ placements are altered accordingly. If not, it differs from the fittest rat’s position. Equation (20) models the GCR movement strategy.

19 $${X}_{i,j}^{new}={X}_{i,j}+C({X}_{K,j}-h{X}_{i,j})$$

20 $${X}_{i}=\left\{\begin{array}{c}{X}_{i,j}+C\left({X}_{i,j}-\alpha {X}_{K,j}\right), {F}_{i}^{new}<{F}_{i}\\ {X}_{i,j}+C\left({X}_{m,j}-\beta {X}_{K,j}\right), otherwise\end{array}\right.$$

where Xi indicates the upcoming state of the ${i}^{th}$ GCR, xnew $i,j$ denotes its value in the dimension $j$, ${X}_{i,j}$ is the present position of GCR, ${X}_{K,j}$ represents the dominant male in the dimension $j$.

Several adjacent locations which may be attained under the impact of Eqs. (21) and (22). Equation (21) describes to imitate the impact of an abundant source of food, which subsequently drives further exploitation. Equation (22) defines $\alpha$ as the coefficient that replicates a dwindling food supply, prompting the search for alternative sources or refuge. Equation (23) defines $\beta$ as the constant amount that leads each rat to relocate its source of nutrition within the refinement region. The parameters $h, \alpha ,$ and $\beta$ are adjusted from the notions in^43^.

21 $$h={F}_{{x}_{k}}-{C}_{iter}(\frac{{F}_{{x}_{k}}}{{Max}_{iter}})$$

22 $$\alpha =\left(2h\right) rand-h$$

23 $$\beta =2h\delta -h$$

where ${C}_{iter}$ is the present iteration, ${Max}_{iter}$ is maximum number of all iterations, ${F}_{{x}_{k}}$ is value of the fitness function corresponding the leading male, ${F}_{{x}_{i}}$ denotes present value of fitness function, $C$ represents an arbitrary factor that is pre-defined, and $h$ represents the effect of a plentiful source of food.

### Exploitation phase

Breeding season changes depending on environment and is generally during the rainy season. During the mating season, males have been seen to disperse from the group. The presumption is that after the group is divided, foraging efforts will be concentrated in locations with plenty of food sources. The phase simulation starts with an arbitrary choice of a female cane rat $f$ such that $f\ne k$ (the leading-male rat). For the reason that refinement happens near plentiful nutrition supplies, strengthening takes place in adjacent to the chosen female. Equation (24) depicts a representation of this stage. If the newly computed GCR location increases the fitness function, as described in Eq. (20), it takes precedence over the previous position.

24 $${X}_{i,j}^{new}={X}_{i,j}+C({X}_{K,j}-\delta {X}_{f,j})$$

where ${x}_{f, j}$ indicates the location of the arbitrarily chosen female, and $\delta$ takes random numbers between 1 and 4 to express the amount of annual generated new generation of youngers. Parameters $C, h, \delta , \rho , \alpha ,$ and $\beta$ improve exploration and exploitation across several repetitions.

**Fig. 7:**
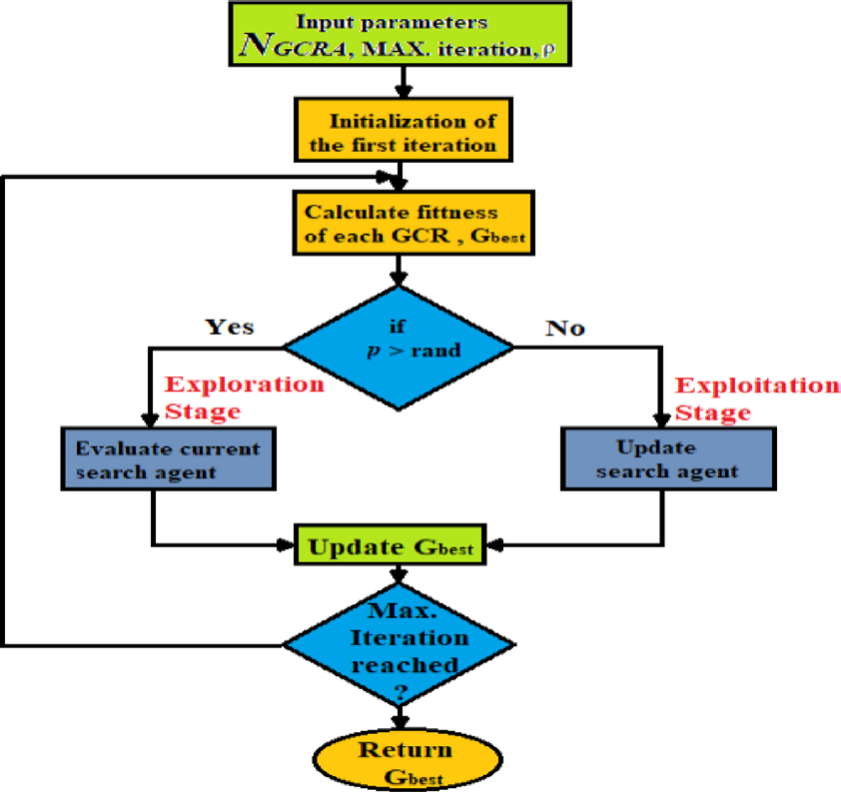
Block diagram of GCRA optimization.

**Fig. 8:**
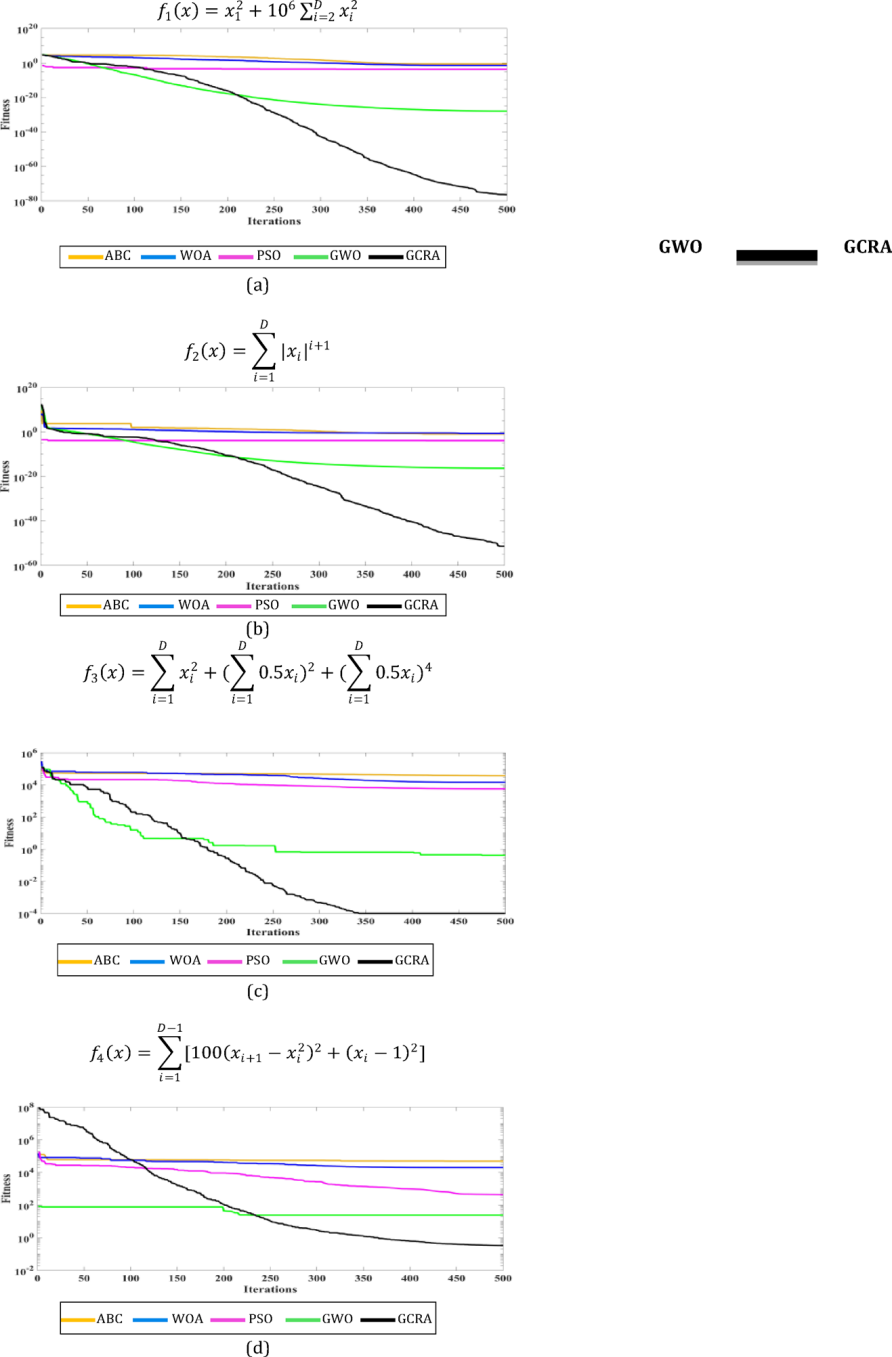
The convergence curves of the four comparative algorithms achieved in some popular benchmark functions.

The position of each GCR is assessed through the fitness function, with the fittest GCR being the one whose position yields the minimum or maximum value of this function. Apart from the initial population generation, the optimization steps are repeated multiple times, as specified by the maximum number of iterations. A summary of GCRA procedures is illustrated in the block diagram in Fig. 7, and flowchart of the proposed GCRA MPPT algorithm is illustrated in Fig. 8.

### Computational complexity

The proposed algorithm is rational and has a less complexity time. The time complexity is evaluated based on the number of greater cane rats (${N}_{GCRA}$), the problem’s dimensions ($d$), the maximum number of iterations (${Max}_{iter}$), Hence, the time complexity of GCRA can be expressed as follows:

25 $${\text{T}}_{{{\text{GCRA}}}} {\text{ = ~T}}_{{{\text{problem~}}\,{\text{definition}}}} {\text{ + ~T}}_{{{\text{Solution}}\,{\text{~creation}}}} {\text{ + T}}_{{{\text{function}}\,{\text{~evaluation~}}}} {\text{ + T}}_{{{\text{update}}\,{\text{solution}}}}$$

The previous equation has the following components for complexity time:

- Problem definition time.
- The GCRA requires (${N}_{GCRA}$× $d$) time to create the random population.
- The function evaluations require (${Max}_{iter}$ ×${T}_{function evaluation}$×${N}_{GCRA}$).
- The time required for solution update is **(**${Max}_{iter}$× ${N}_{GCRA}$× $d$).

Therefore, based on the previous components, Eq. (25) can be simplified approximately to formula in Eq. (26):

26 $${\text{T}}_{{{\text{GCRA}}}} \cong {\text{Max}}_{{{\text{iter}}}} *{\text{N}}_{{{\text{GCRA}}}} *~{\text{T}}_{{{\text{function~evaluation~}}}} + {\text{Max}}_{{{\text{iter}}}} *d$$

From the previous equation, it is clear that the GCRA algorithm has a linear time complexity. Which means that the GCRA is considered a computationally efficient algorithm.

Comparing to other optimization algorithms such as gray wolf optimization (GWO) and particle swarm optimization (PSO), they share some similarities and some differences. For GWO, the computational complexity depends on number of wolves (${N}_{W}$), dimension of problem (*D*), cost of objective function (${C}_{f}$), and the number of iterations (*T*). The overall complexity consists of that related to population sorting $O\left(N.Log N\right)$ and the other related to position update $O(N.D)$.The overall complexity is expressed in Eq. (27)

27 $$O= T.((N.\text{log}N)+N.D))$$

Regarding the PSO, its overall complexity is influenced by factors of number of particles (${N}_{P}$), problem`s dimensionality (*D*), and iteration number (*T*). The overall complexity consists of the fitness evaluation $O\left(N*f\left(D\right)\right)$, velocity and position update $O(N*D)$, and the update of global best $O\left(N\right)$. Thus, the overall complexity can be described in Eq. (28)

28 $$O= T.(\left(N.D\right)+(N.f\left(D\right))$$

However, these similarities, but due to the specific mechanism and strategies employed by each one of these algorithms, this may lead to differences in both the complexity burden and solution quality.

Particularly, in real-world implementation, the complexity may need to be reduced, but this could lead to a decrease in solution accuracy. In other words, there is a trade-off between the algorithm’s complexity and the accuracy of the solution. Therefore, these parameters must be chosen carefully.

For more evaluation of the complexity and convergence of the GCRA algorithm in comparison to other meta-heuristic optimization techniques, a set of benchmark test functions is applied to GCRA as well as algorithms like PSO, GWO, WOA, and ABC. The relationship between fitness values and the number of iterations for each algorithm is depicted in fitness function obtained values in Table 1.

**Table 1:** The best fitness functions obtained by algorithms.

Test functions	Best objective function obtained				
	ABC	WOA	PSO	GWO	GCRA
${f}_{1}\left(x\right)={x}_{1}^{2}+{10}^{6}\sum_{i=2}^{D}{x}_{i}^{2}$	$2.4*{10}^{-1}$	$4.2*{10}^{-2}$	$2.66*{10}^{-4}$	$1.14*{10}^{-28}$	$4.87*{10}^{-77}$
${f}_{2}\left(x\right)=\sum_{i=1}^{D}{|{x}_{i}|}^{i+1}$	$1.41*{10}^{-1}$	$2.62*{10}^{-1}$	$1.48*{10}^{-4}$	$5.41*{10}^{-17}$	$4.1*{10}^{-52}$
${f}_{3}(x)=\sum_{i=1}^{D}{x}_{i}^{2}+{(\sum_{i=1}^{D}0.5{x}_{i})}^{2}+{(\sum_{i=1}^{D}0.5{x}_{i})}^{4}$	$3.7*{10}^{4}$	$1.46*{10}^{4}$	$5.79*{10}^{3}$	0.42	$1.2*{10}^{-4}$
${f}_{4}(x)=\sum_{i=1}^{D-1}[100{\left({x}_{i+1}-{x}_{i}^{2}\right)}^{2}+{({x}_{i}-1)}^{2}]$	$4.9*{10}^{4}$	$2.1*{10}^{4}$	443.6	24.35	0.33

**Fig. 9:**
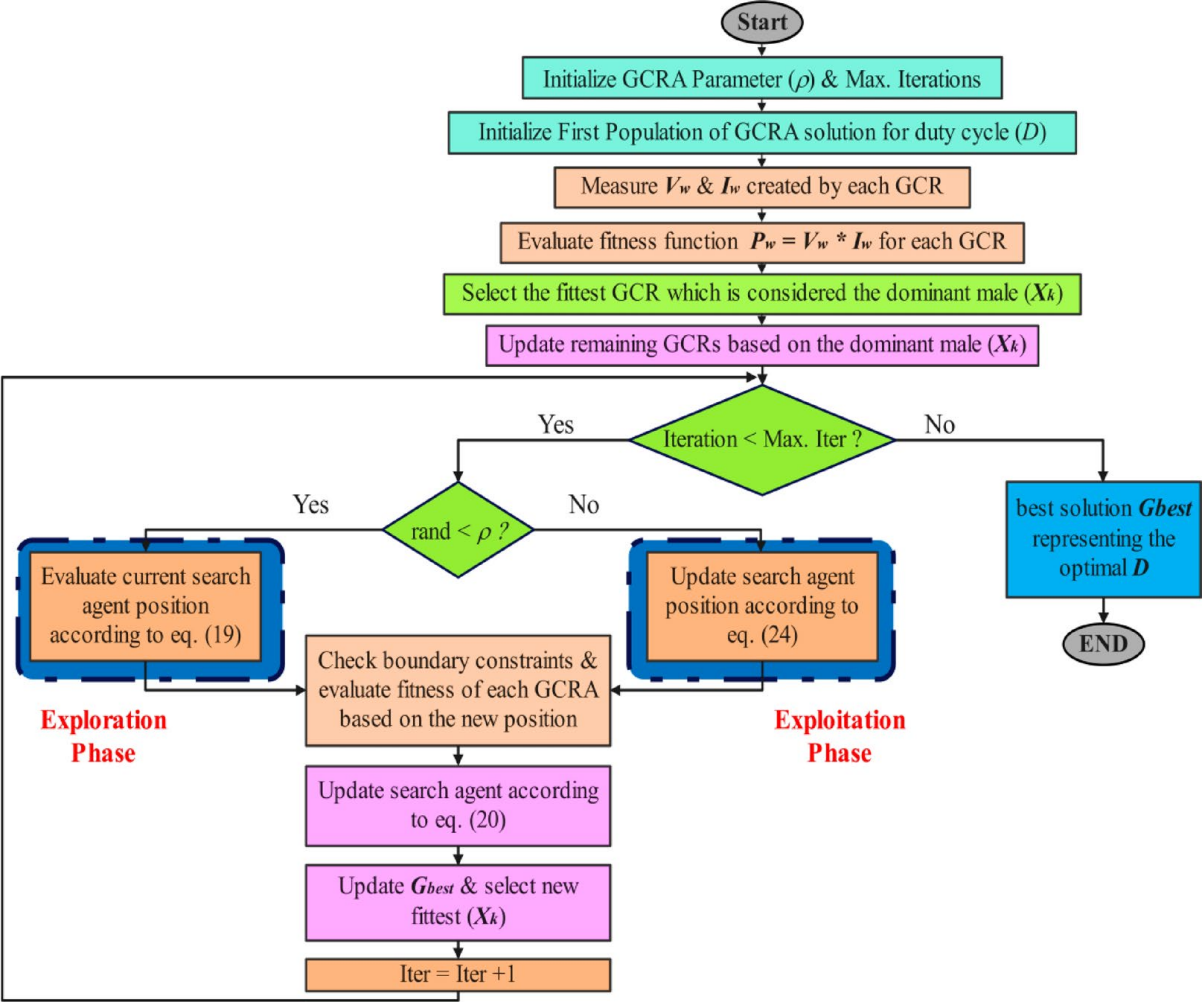
Flowchart of the proposed GCRA-based MPPT.

The correspondent convergence curves shown in Fig. 9.

Key advantages and limitations of the proposed algorithm compared to other MPPT techniques are summarized in Table 2.

**Table 2:** Detailed comparison of different MPPT methods.

Algorithm	Exploration and exploitation	Tracking efficiency	Oscillation at steady state	Convergence speed	Computational complexity	Robustness
PSO^44^	Good balance, but can get stuck in local optimal	High	Medium	High	High	Medium
GWO^45^	Strong exploration, moderate exploitation	High	Medium	High	Medium	High
BA^46^	Strong exploration, weak exploitation	High	Medium	High	High	High
ABC^47^	Strong exploration, require tuning	High	Low	Medium	High	Medium
ACO^48^	Strong exploration, require tuning	Medium	Low	Medium	Medium	Medium
WOA^49^	Good balance, but can get stuck in local optimal	High	Medium	Medium	High	High
GCRA	Balanced with adaptive strategy	High	Low	High	Medium	High

## Results and discussion

The simulation results provide insight into effectiveness and robustness of the proposed methodology of GCRA-based MPPT optimization strategy. Simulation is performed in MATLAB/SIMULINK R2022b using a discrete solver with a sampling period of 2.5-μ seconds. Simulations are carried out on a stand-alone wind system consisting of a permanent magnet synchronous generator (PMSG) which is derived directly by a small wind turbine. The parasitic characteristics of both the wind turbine and the PMSG are presented in Tables 3 and 4. Also, the parameters of dc-dc boost converter are illustrated in Table. 5.

**Table 3:** Parameters of the wind turbine.

Parameter	Value
Rated mechanical output power ($${P}_{m})$$ ($$kW)$$	12
Base wind speed ($$m/s)$$	12
Total mechanical inertia ($$J$$) ($$kg.{m}^{2})$$	800
The air density ($$\rho$$) ($$kg/{m}^{3})$$	1.08

**Table 4:** Parameters of permanent magnet synchronous generator.

Parameter	Value
Number of phases	3
Type of generator’s rotor	Salient pole
Phase resistance of stator (${R}_{s}$) (*Ω*)	0.0485
Direct-axis synchronous inductance ${L}_{d}(H)$	395*${10}^{-6}$
Quadrature-axes synchronous inductance ${L}_{q}(H)$	395*${10}^{-6}$
Flux linkage (${w}_{b}$)	0.1194

**Table 5:** Parameters of DC-DC boost converter.

Parameter	Value
Switching Frequency ($${f}_{s}$$) ($${kH}_{z})$$	20
Inductor ($L$) ($\mu H)$	45.57
Capacitor ($C$) ($\mu F)$	665.625
Minimum Output Voltage ($V$)	50
Maximum Output Voltage ($V$)	400
Load Resistance ($R$) (*Ω*)	13.33

Two distinct test cases are investigated for endorsement. The first one shows step variations of the wind velocity, and the second case examines the impact of a realistic changing random wind speed profile developed by accounting for the nonlinear characteristics of winds. In both scenarios, the wind speed is simulated between 6 and 12 m/s based on data mentioned in^10^,^12^,^22^. In addition, the system is tested utilizing several MPPT methodologies based on other optimization techniques such as PSO, GWO, and conventional P&O under the two test scenarios. All findings were compared in both instances to illustrate the effectiveness and efficiency of all approaches under consideration. A sensitivity analysis of GCRA^37^ was performed, demonstrating the influence of key parameters on the algorithm’s performance. For example, factors such as the exploration rate, population size, and number of iterations significantly impact the results. A higher exploration rate can enhance accuracy but may also slow down the system and introduce oscillations. Based on the parametric analysis, the optimal values were determined as 0.5 for the exploration rate, 10 for the population size, and 100 for the number of iterations to achieve a balance between accuracy and system stability. Also, the upper and lower bounds of the search space have also been clearly defined: the duty cycle is constrained between 0 and 1 to ensure that the optimization remains within the physical and safe operating limits of the system. Values of parameters used in each considered algorithm is illustrated in Table 6.

**Table 6:** Parameters of MPPT algorithms.

GCRA		GWO		PSO		P & O	
$\rho$	0.5	${r}_{1}$	1	$W$	1	${D}_{init}$	0.4
$\delta$	[1 4]	${r}_{2}$	1	${C}_{1}$	1.5	$dD$	0.01
C	[0 1]	* − *	–	${C}_{2}$	2	–	–
${N}_{GCRA}$	10	${N}_{p}$	6	${N}_{PSO}$	10		
$Max.$ $Iteration$	100		100		100	–	–

### Case 1: step variation of wind speed

**Fig. 10:**
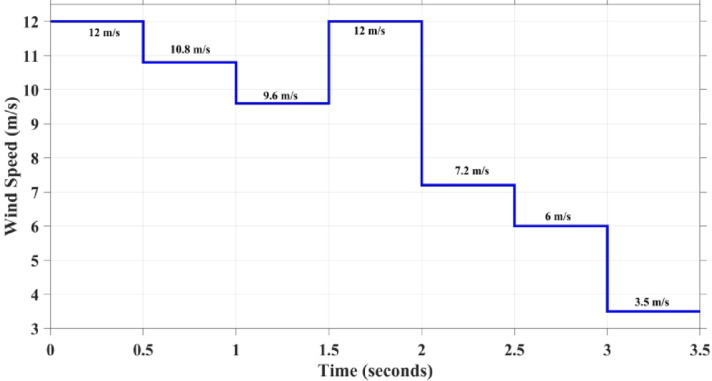
Wind speed pattern with step variations.

**Fig. 11:**
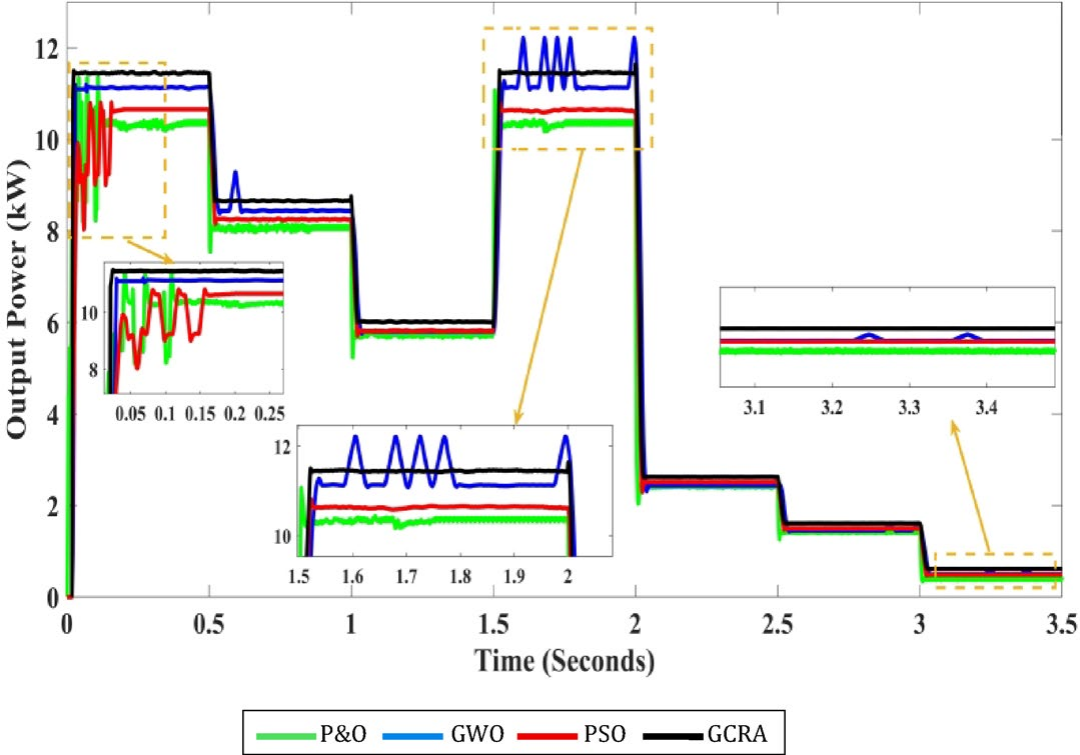
The output power of each MPPT considering step variation of speed of wind.

To evaluate the proposed technique, step variation of wind speed pattern in between a very low speed near the cut-in speed of 3.5 and 12 m/s are generated, as shown in Fig. 10. All MPPT techniques are compared together according to their efficiency, oscillation ratio, and Cp change. The correspondent output power for each concerned technique is shown together in Fig. 11 Which indicates the output power of wind system at each wind speed utilizing different MPPT techniques. It is worth to mention that the tracking efficiency of each MPPT can be calculated using the following equation:

29 $$\eta _{{{\text{MPPT}}}} = \frac{{\mathop \smallint \nolimits_{0}^{{T_{{{\text{sim}}}} }} P_{{{\text{act}}}} }}{{\mathop \smallint \nolimits_{0}^{{T_{{{\text{sim}}}} }} P_{{{\text{Theoretical}}}} }}$$

where ${P}_{act}$ is the actual power output to the load, and ${P}_{theoritical}$ is the theoretical optimal output power to the load if the MPPT has a theoretical tracking efficiency of 100%.

From Fig. 11, it is shown that the best tracking technique is the proposed GCRA, as its output power is the highest one among the others for the first four periods (i.e., high and medium wind speed). Followed by GWO and then PSO, finally the conventional P&O technique. On the other hand, for the last two simulation periods (i.e., low wind speed), the extracted output power from the system is almost the same for all compared techniques. However, the proposed GCRA-based MPPT strategy shows a slight improvement (i.e., 2.55 kW and 1.6 kW for the wind speeds 7.2 m/s and 6 m/s, respectively). In addition, it is shown in the figure that the oscillation of the power at steady state is very low (i.e., less than 1%) under techniques of conventional P&O, PSO, and proposed GCRA. On the other hand, the power oscillation under GWO is higher which may reach to about 9.7% causing power loss in the system. Table 7 presents the theoretical output power, including both the mechanical power from the turbine and the electrical power from the PMSG, as well as the optimal power delivered to the load while accounting for efficiency and losses at each stage. Additionally, the table displays the actual power output to the load when using the various MPPT algorithms under evaluation.

**Table 7:** The output Power for each MPPT Technique at steady-State.

Wind speed (m/s)	${P}_{mech}$ output from wind turbine (kW)	${P}_{elec}$ output from PMSG (kW)	Optimal output power to the load (kW)	Actual output power (kW)			
				P&O	PSO	GWO	GCRA
12	12	11.7	11.5	10.3	10.8	11.1	11.45
10.8	9.2	8.9	8.7	8	8.3	8.4	8.6
9.6	6.6	6.45	6.3	5.7	5.8	5.8	6.2
12	12	11.7	11.5	10.3	10.8	11.1	11.45
7.2	2.7	2.63	2.6	2.4	2.5	2.45	2.55
6	1.7	1.66	1.6	1.45	1.5	1.48	1.58
3.5	0.7	0.68	0.66	0.57	0.58	0.58	0.64

**Fig. 12:**
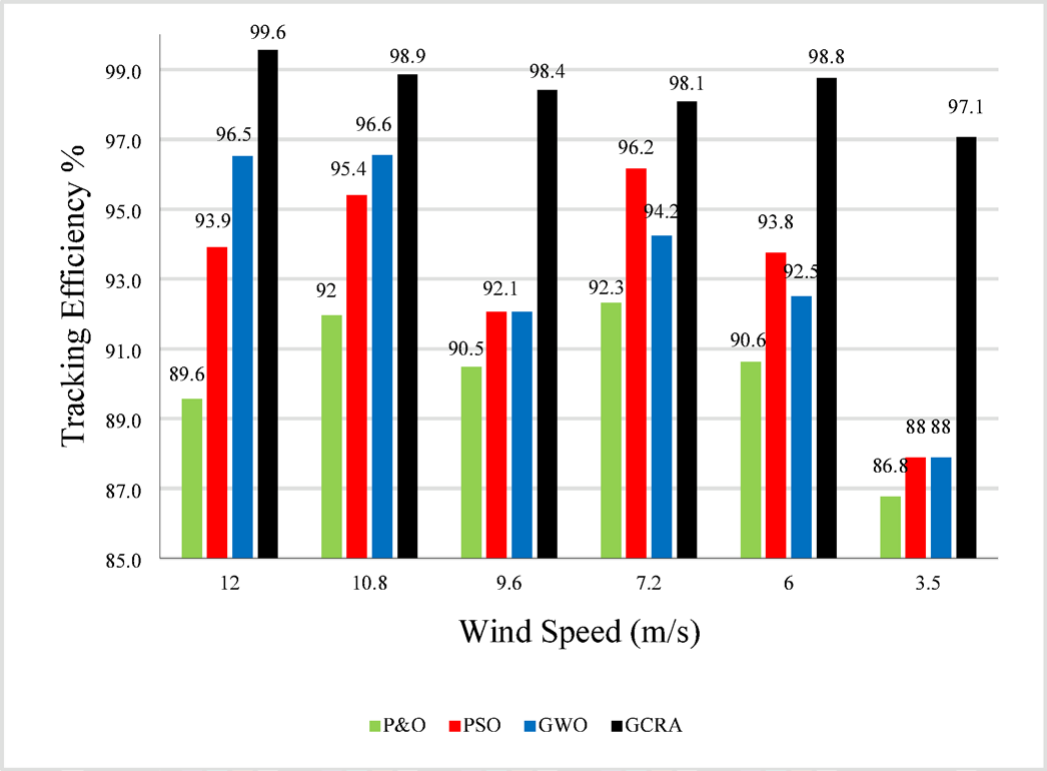
The output power tracking efficiency for the four different methodologies at several wind speeds.

Tracking efficiency for each technique under deferent wind speed is illustrated in Fig. 12.

**Fig. 13:**
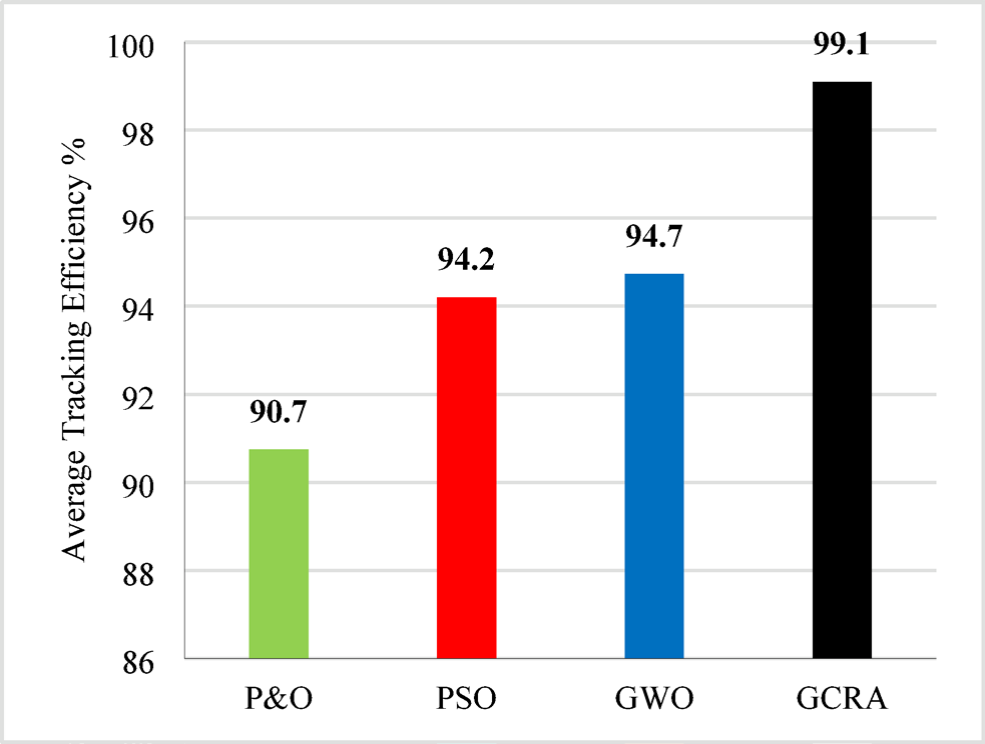
The output power’s average tracking efficiency over the whole period for the 4 different methodologies considering multi-step wind speed pattern.

For overall evaluation of the performance of each technique under the step pattern of wind speed, the average efficiency of each technique under the whole variation time is calculated and shown in Fig. 13. From the figure, it is clear that the proposed GCRA has the highest average tracking efficiency by 99.1% followed by GWO by 94.7% and PSO by 94.2% and conventional P&O by 90.7%.

**Fig. 14:**
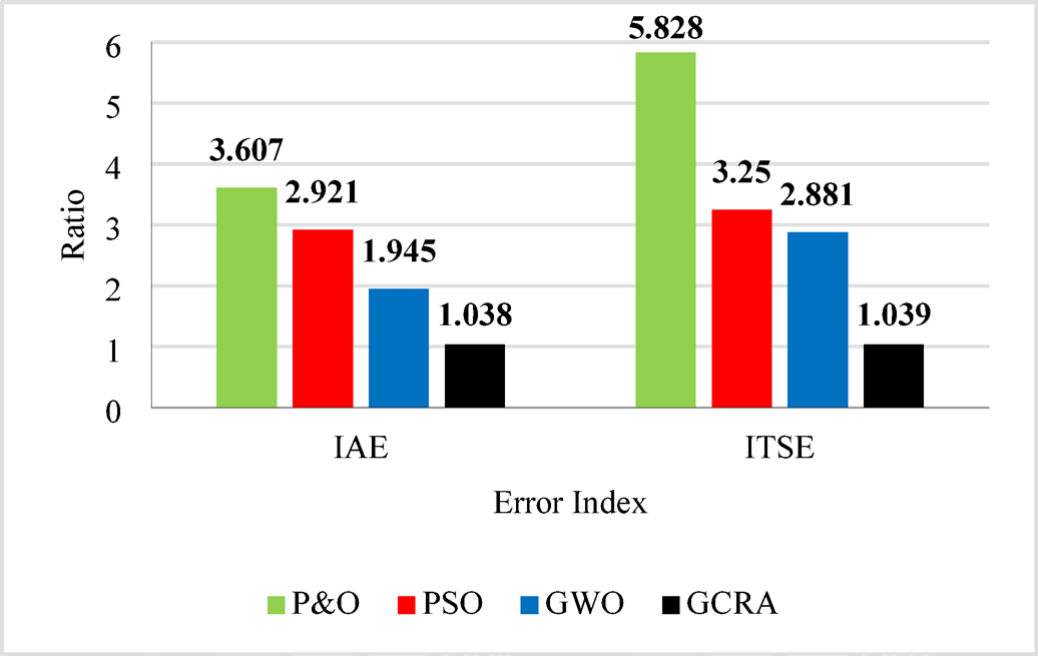
Control performance indices for output power under step variation of wind speed.

Furthermore, there are numerous performance indices for current recent closed-loop control systems to measure the sensitivity and precision, as well as tracking performance^40^,^41^. These indices directly indicate qualities such as overshoot, speed of controller`s response, and time of settling. These indices performance evaluation include integral absolute error (IAE) and integral time square error (ITSE), which may be calculated using the following Equations:

30 $$IAE=\underset{0}{\overset{{T}_{sim}}{\int }}\left|e\left(t\right)\right|.dt$$

31 $$ITSE=\underset{0}{\overset{{T}_{sim}}{\int }}t.\left|{e}^{2}\left(t\right)\right|.dt$$

where $e(t)$ is the error between the theoretical power and the actual output power, and ${T}_{sim}$ is the simulation time. By applying these performance evaluation criteria here, both IAE and ITSE values for all utilized algorithms are illustrated in Fig. 14. It is clear that from Fig. 12, the best one is the proposed GCRA which has the least values of indices by about 1 for both IAE and ITSE indices, followed by GWO by 1.945 and 2.88 for IAE and ITSE, respectively. In the third rank, PSO-based MPPT produces error with indices 2.92 and 3.25 for IAE and ITSE, respectively. Conventional P&O-based MPPT generates the largest error by indices 3.61 for IAE and 5.83 for ITSE.

**Fig. 15:**
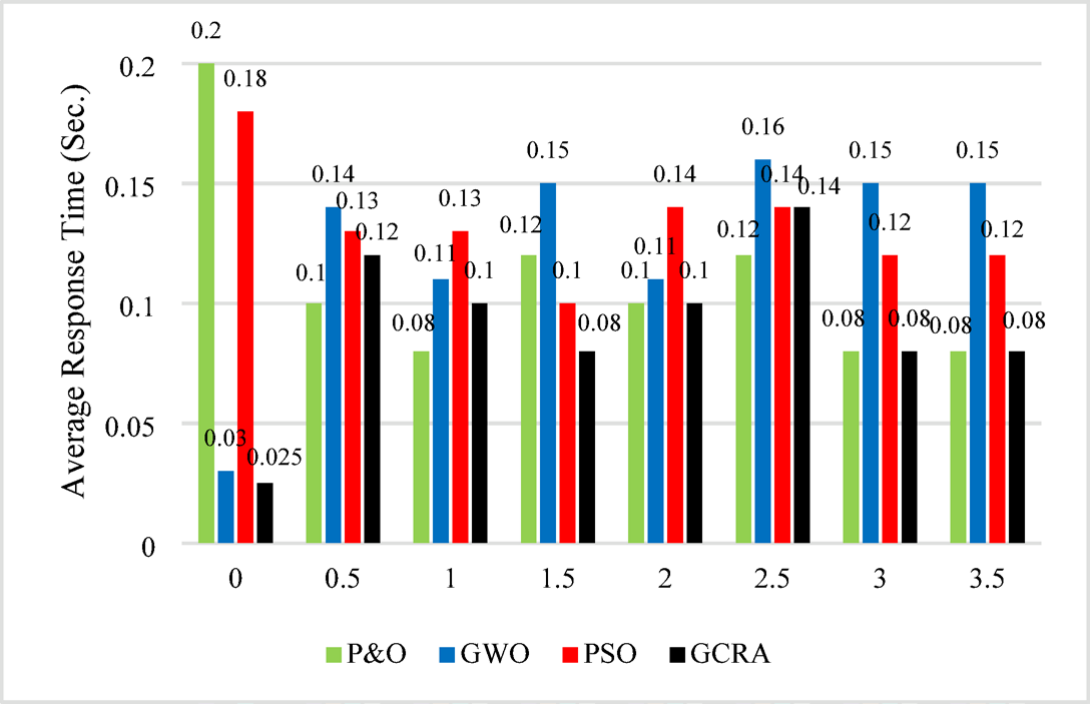
Average response time at each step of variation for all methodologies.

**Fig. 16:**
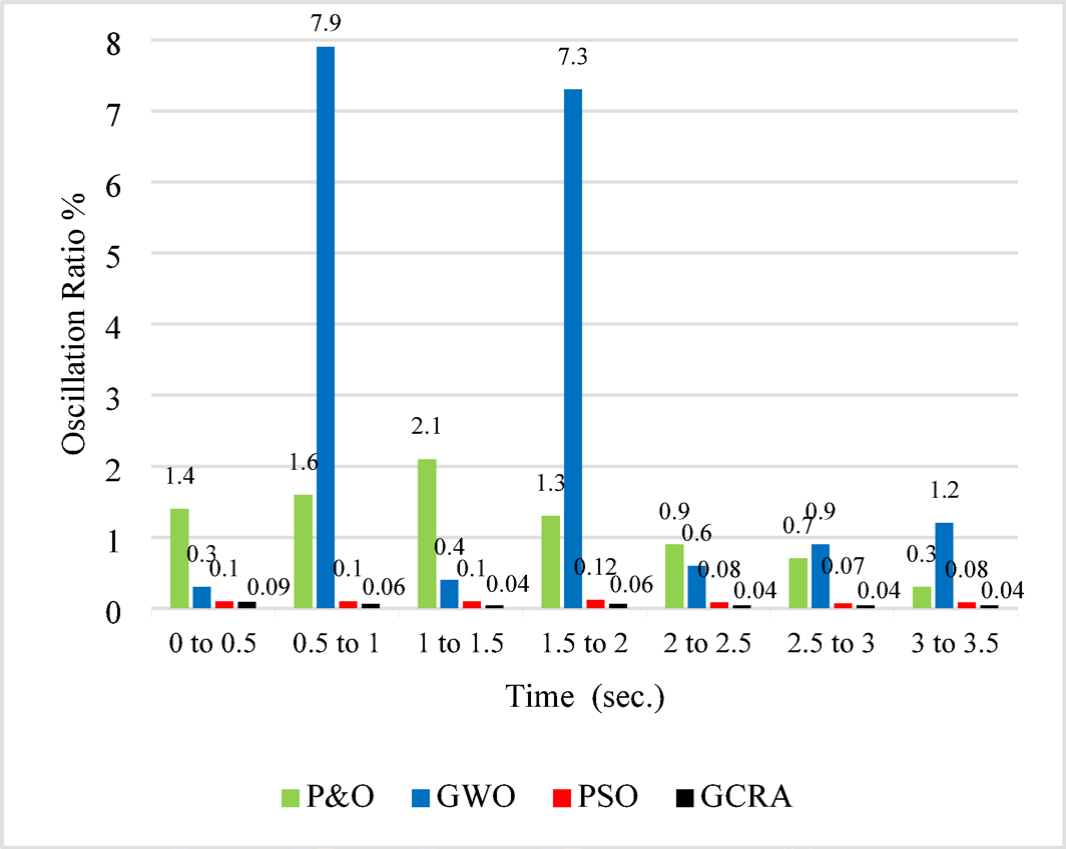
Average Oscillation ratio at each step of variation for all methodologies.

Besides, the tracking efficiency, the response time and oscillation ratio are key factors in assessing the controller’s performance. Therefore, Figures 15 and 16 provide a detailed analysis of the oscillation ratio and response time, respectively.

From the figure, it is evident that the proposed GCRA exhibits the shortest response time. While P&O may occasionally respond faster, GCRA remains superior as it achieves MPP with the highest efficiency and the lowest oscillation ratio, as illustrated in Figure 16. This figure provides a detailed analysis of the oscillation ratio at different time intervals. Although GWO demonstrates high efficiency, as previously discussed, it experiences a significantly higher oscillation ratio compared to the other methods. Conversely, GCRA maintains the lowest oscillation ratio throughout the entire simulation period, never exceeding 0.1%. This highlights its robustness, ensuring the highest tracking efficiency, the fastest response time, and minimal oscillations.

**Fig. 17:**
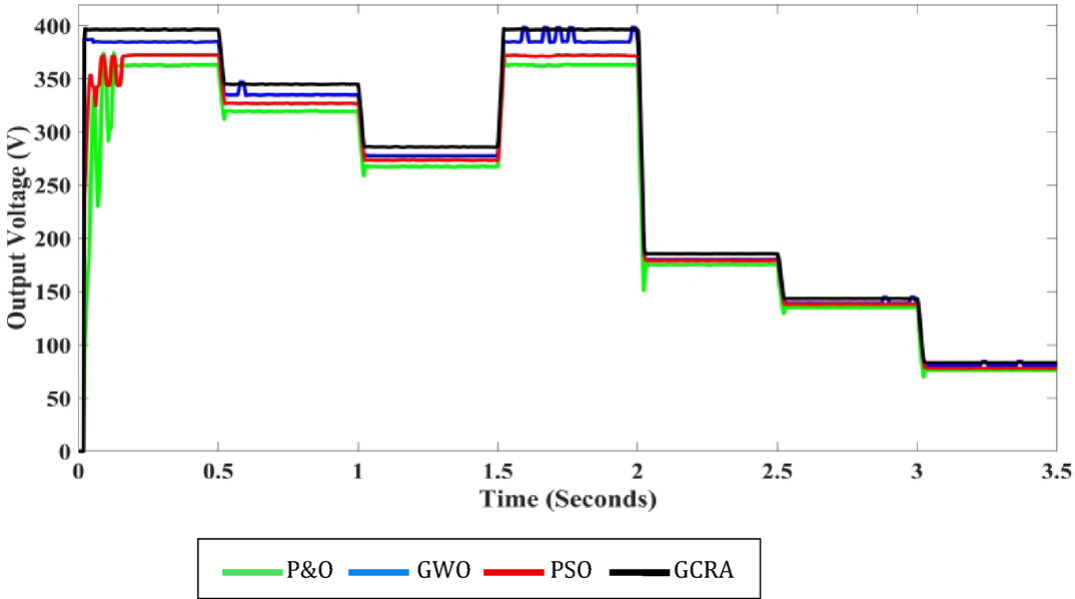
Output voltage profile for each MPPT technique under step variation of wind speed.

For additional comparison purposes, the output voltage profile for each approach is provided in Fig. 17, which exhibits roughly the same patterns as the corresponding power profile. The suggested GCRA generates the maximum ripple-free voltage.

**Fig. 18:**
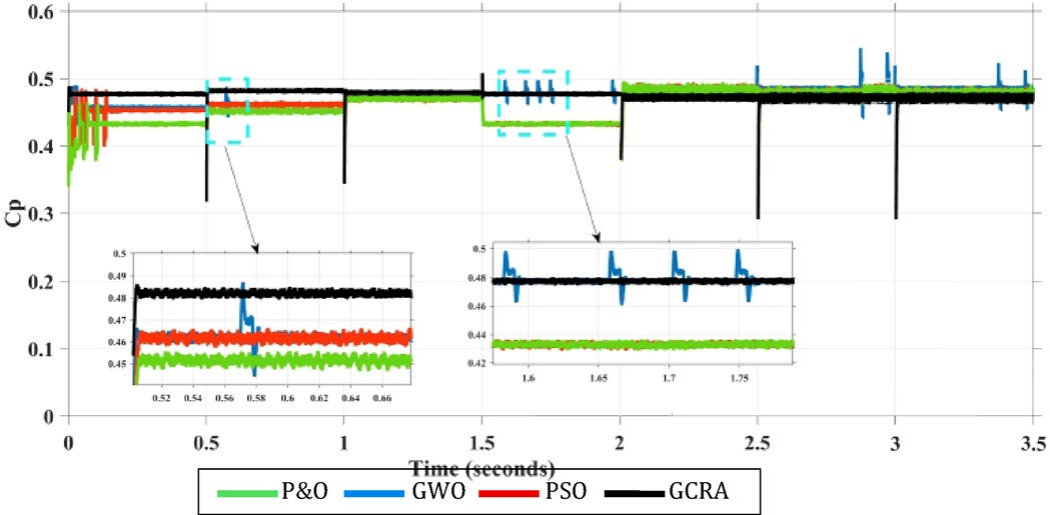
Variation of turbine power coefficient ${C}_{p}$ under step wind pattern for each MPPT technique.

Furthermore, comparisons must include the power coefficient (Cp) of each approach to study its ability to exploit the wind power. Variation of the power coefficient over the entire test period is shown in Fig. 18. From the figure, it is clear that among all techniques, the proposed GCRA-based MPPT has the maximum value of Cp equals 0.48 under different wind speed which is the optimal Cp value of wind turbine when *β* equals zero, as mentioned before. This means the optimal utilization of wind power is achieved under all values of wind speed. Also, From the zoomed portions, it is illustrated that proposed technique has the least Cp ripples by about 0.875%. On the other hand, other techniques have varying Cp coefficient which means that utilization of the turbine power is not optimal. Also, ripples of Cp with 4.52% for GWO, 1.44% for PSO, and 1.64% for conventional P&O which leads to some power losses in turbine power.

### Variation of turbine power coefficient ${C}_{p}$ under step wind pattern for each MPPT technique.*Case 2: turbulent Variation of Wind Speed*

**Fig. 19:**
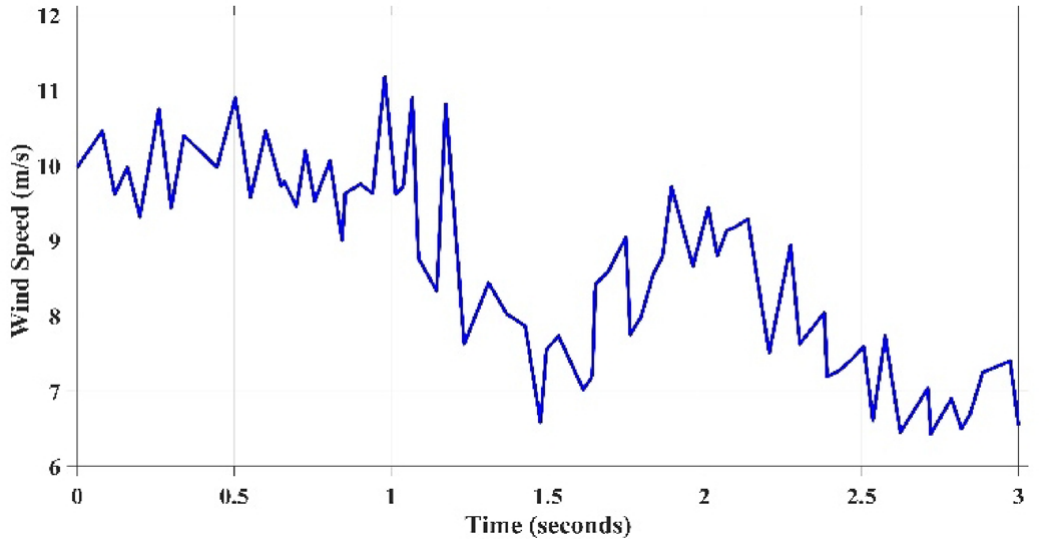
Wind speed pattern with turbulent variations.

**Fig. 20:**
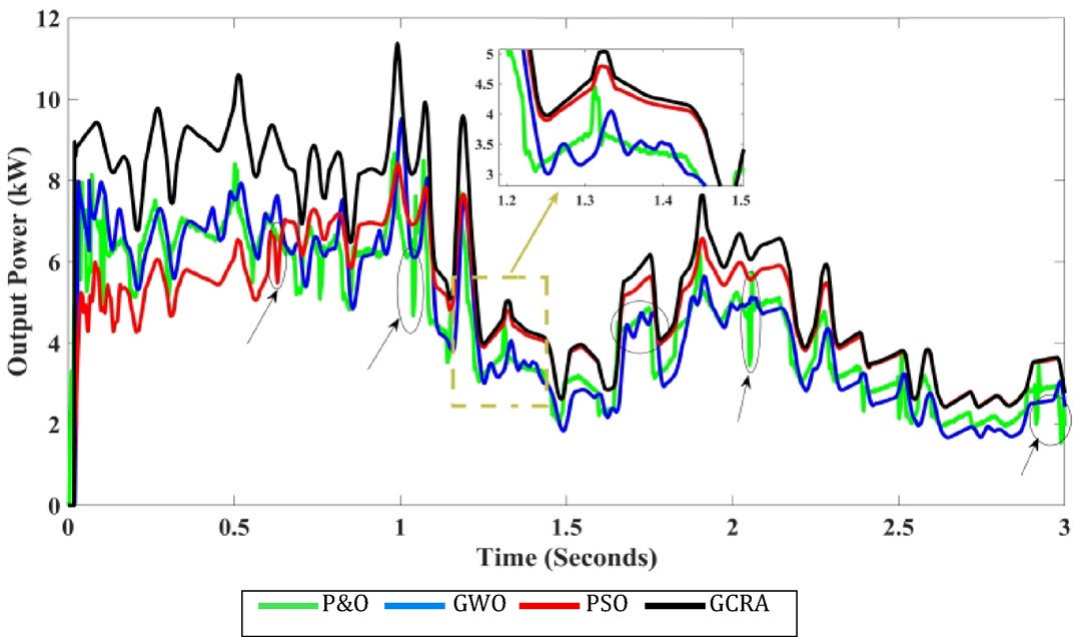
The output power for each MPPT considering turbulent variation of wind speed.

**Fig. 21:**
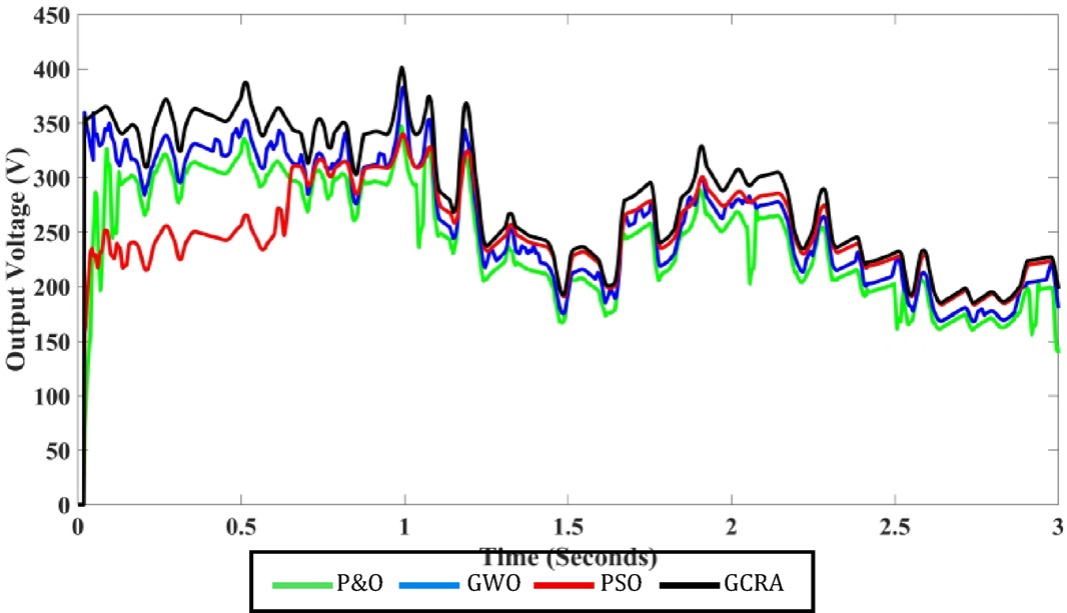
The output voltage for each MPPT considering turbulent variation of wind speed.

Till now the performance of all techniques are tested under step variation of wind speed. Nevertheless, in the real world the wind speed varying rapidly and randomly. Therefore, all these techniques will be tested again under a realistic pattern of wind. This scenario evaluates the performance of all considered MPPT approaches to be assessed under a realistic variation spanning from the speed of wind equals 6 m/s to the speed equals 12 m/s, as shown in Fig. 19. It is clearly evident that wind speed fluctuates regularly over the whole period of system operation. The developed algorithms are assessed based on the output electrical power that can be extracted from WECS and also its output voltage. The correspondent output power and voltage for each concerned technique is shown in Figs. 20 and 21, respectively. The obtained curves show that the proposed GCRA-based MPPT has the prefect tracking for the realistic pattern of the wind speed as it has the exact profiles of the wind pattern with very low voltage ripples and approximately zero steady-state inaccuracy. On the other hand, the other utilized MPPT techniques have some ripples and errors in both power and voltage profiles. Moreover, the proposed GCRA has the largest extracted output power over the entire period among the others.

**Fig. 22:**
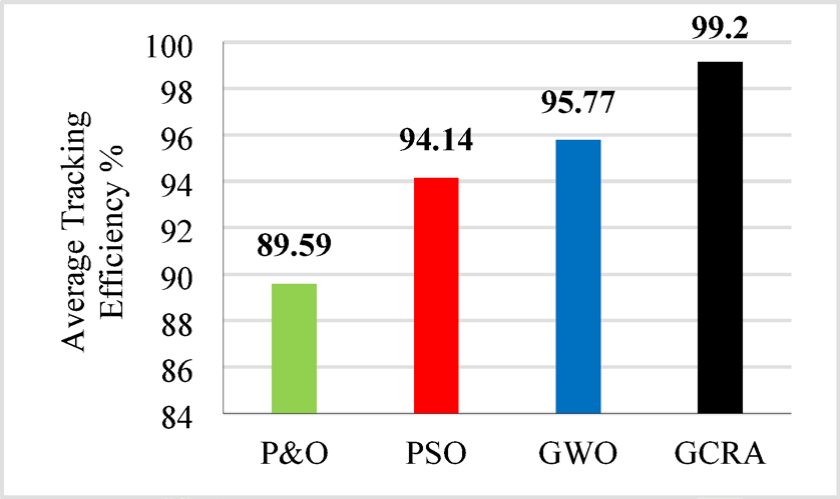
The output power’s average tracking efficiency over the whole period for the 4 different methodologies considering realistic turbulent wind speed pattern.

The average efficiency of all investigated techniques under study is compared according to the average generated output power which can be illustrated in Fig. 22. Which shows that the best power tracking efficiency is achieved by GCRA by 99.2% followed by GWO by 95.8% and PSO by 94.1, and the least average efficiency is accomplished by conventional P&O by 89.6%.

From all the above, it is worth to mention that the proposed GCRA-based MPPT technique inherently addresses dynamic conditions through its exploration–exploitation balance, which enables rapid adaptation to wind speed variations without requiring a separate reinitialization strategy. Specifically, the population update mechanism allows candidate solutions to continuously track changes in the operating point, while the adaptive fitness evaluation (i.e., instantaneous power) ensures that the search converges toward the new global maximum whenever conditions change.

Additionally, the customized boundary handling ensures that updated solutions remain within feasible ranges of duty cycle, preventing abnormalities during fast transients. The simulation results under step and turbulent wind profiles confirm that the proposed approach maintains robustness and stable convergence under dynamic operating conditions.

### Case 3: ramp gradual variation of wind speed

**Fig. 23:**
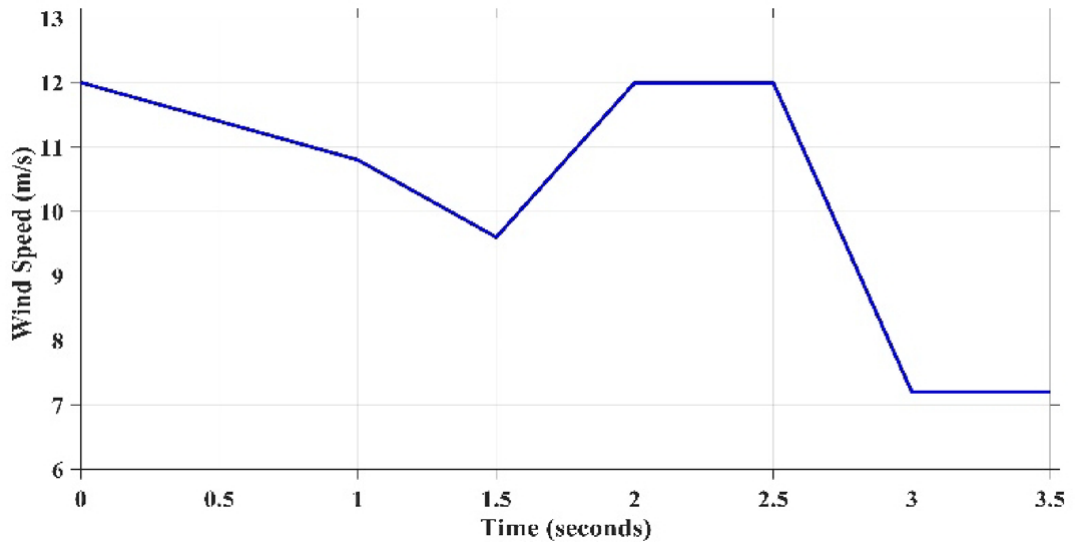
Wind speed pattern with ramp gradual variations.

**Fig. 24:**
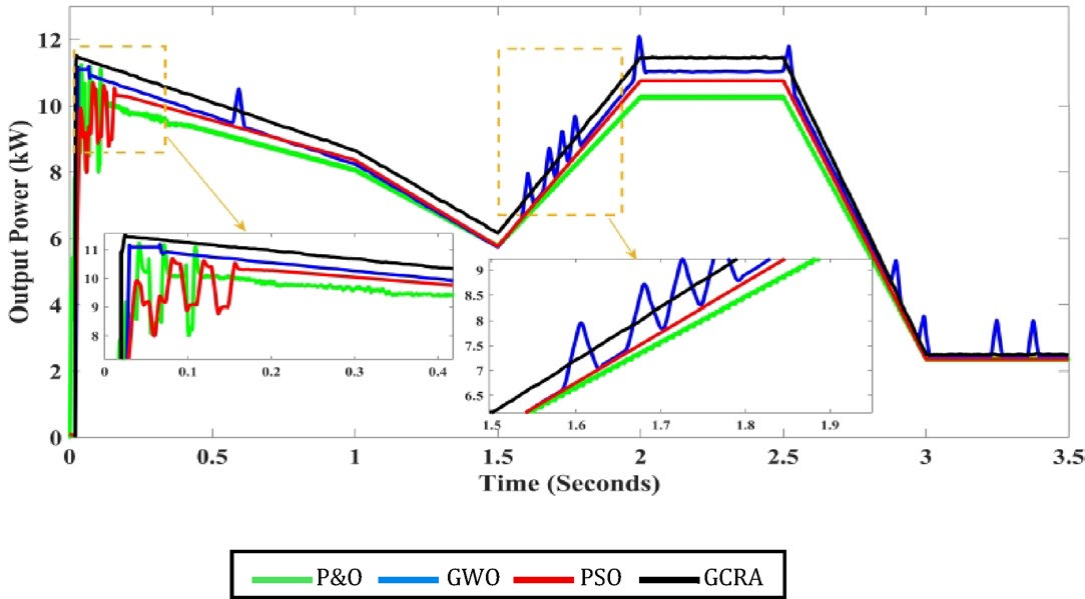
The output power for each MPPT considering ramp variation of wind speed.

The proposed technique is further evaluated and benchmarked against alternative methods under a ramp variation of wind speed, as illustrated in Fig. 23. This profile provides a realistic representation of wind speed dynamics. The corresponding output power of the WECS obtained using each MPPT technique is presented in Fig. 24.”

**Fig. 25:**
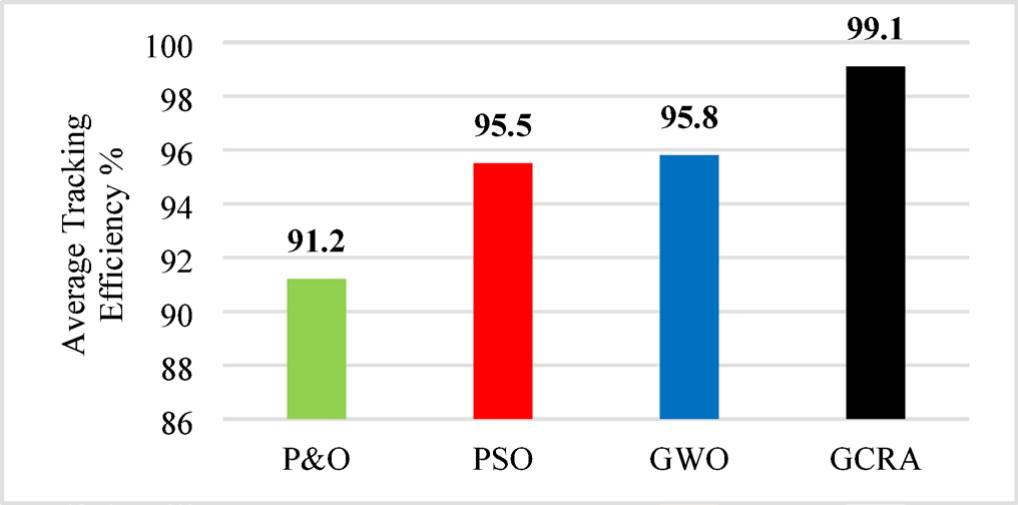
The output power’s average tracking efficiency over the whole period for the 4 different methodologies considering ramp gradual wind speed pattern.

As illustrated in the previous figure, the proposed GCRA demonstrates the highest tracking efficiency, achieving an average of 99.1% compared to 95.5% for PSO, 95.8% for GWO, and 91.2% for P&O. In terms of tracking speed and steady-state response, GCRA exhibits the fastest convergence, reaching stability within 0.04 s, whereas GWO, P&O, and PSO require 0.09 s, 0.15 s, and 0.18 s, respectively. Furthermore, with respect to oscillatory behavior, both GCRA and PSO exhibit negligible oscillations, while P&O shows a noticeable oscillation level, and GWO experiences the most pronounced oscillations among the tested methods. The average efficiency of all investigated techniques can be illustrated in Fig. 25.

To summarize all the obtained results to evaluate the proposed algorithm against other existing considered techniques, Table 8 summarizes all these numerical results.

**Table 8:** Summary Comparison of considered MPPT algorithms.

Algorithm	Average Tracking efficiency (%)			${C}_{p}$ ripples $(\%)$	Error indices	
	Step wind speed variation	turbulent wind speed variation	ramp wind variation		IAE	ITSE
P&O	90.7	89.59	91.2	1.64	3.607	5.828
PSO	94.2	94.14	95.5	1.44	2.921	3.25
GWO	94.7	95.77	95.8	4.52	1.945	2.881
GCRA	99.1	99.2	99.1	0.875	1.038	1.039

## Conclusion

The importance of enhancing the efficiency and performance of wind turbine systems cannot be overstated. Achieving maximum power point tracking (MPPT) under varying wind speeds is particularly challenging due to the complex and random nature of wind. This study addresses this challenge by employing the Greater Cane Rat Algorithm (GCRA), a sophisticated approach aimed at optimizing the MPPT process with low complexity. In this research, the proposed MPPT technique was implemented using MATLAB/Simulink for an off-grid wind turbine system with a permanent magnet synchronous generator, forming a Wind Energy Conversion System (WECS). Notably, the approach eliminates the need for additional mechanical sensors, thus reducing implementation costs. Comparative analysis with other MPPT techniques such as Perturb and Observe (P&O), Particle Swarm Optimization (PSO), and Grey Wolf Optimization (GWO) revealed that the proposed GCRA-based approach outperformed traditional methods. Specifically, it achieved impressive tracking efficiencies exceeding 99% across various wind speed scenarios, with minimal oscillations and negligible error indices (i.e., IAE and ITSE around 1). Other MPPT algorithms, on the other hand, only obtained average tracking efficiencies of 94.7%, 94.2%, and 90.7%, as well as greater error indices of 1.945, 2.92, and 3.61, respectively.

In general, this research underscores the potential of the GCRA-based MPPT approach to enhance efficiency and reliability in wind energy systems, paving the way for further advancements in renewable energy technology. Although the proposed GCRA-based MPPT approach may present certain limitations, the simulation results demonstrate that these challenges can be effectively mitigated. One concern is the need for multiple iterations to achieve convergence, which may lead to higher computational demands compared to lightweight methods such as Perturb and Observe (P&O). However, the obtained results highlight the superior efficiency of GCRA over other algorithms despite this drawback.

Furthermore, while simulations yielded valuable insights, several limitations were acknowledged, including the absence of experimental validation. Hardware implementation provides an opportunity to explore challenges and limitations that may not appear in simulations. These include sensor accuracy and noise which may cause suboptimal power tracking. Additionally, power converter losses which must be considered in real world, response time and control delays of digital controllers such as DSP, FPGA, or microcontrollers may slow tracking speed particularly under rapidly changing conditions. Also, this work focuses solely on a wind turbine with a fixed pitch angle.

These limitations suggest several promising avenues for future research directions include applying experimental prototype to validate the simulation findings, exploring the impact of variable pitch angles on MPPT performance, evaluating the approach in on-grid systems, addressing hybridization between the considered algorithm in this work and any recent algorithm to examine the probabilities of performance improvement. Further, extending the proposed technique to be applied to other renewable energy sources.

## Data Availability

The authors confirm that the data supporting the findings of this study are available within the article.

## Abbreviations

WECS: Wind energy conversion system
P&O: Perturb and observe
MPPT: Maximum power point tracking
GCRA: Greater cane rat algorithm
PMSG: Permanent magnet synchronous generator
PSO: Particle swarm optimization
GWO: Grey wolf optimization
RESs: Renewable energy sources
DFIG: Doubly-fed induction generator
SCIG: Squirrel cage induction generator
BTBC: Back-to-back converter
ORB: Optimum relation based
TSR: Tip speed ratio
PSF: Power signal feedback
ACO: Ant colony optimization
ABC: Artificial bee colony
OTC: Optimal torque control
IC: Incremental conductance
ANN: Artificial neural network
ANFIS: Adaptive neuro-fuzzy inference system
C-SMC: Conventional sliding mode controller
SO-SMC: Second-order sliding mode controller
IPCs: Indirect power controllers
DPC: Direct power control
MPP: Maximum power point
PI: Proportional integral
GCR: Greater cane rat
LB: Lower bound
UB: Upper bound
WT: Wind turbine
BA: Bat algorithm
WOA: Whale optimization algorithm

## References

[1] Navidi, A. & Khatami, F.A.-S. Energy management and planning in smart cities. *CIRED–Open Access Proc. J.* **207**, 2723–2725 (2017).

[2] Kumar, M. et al. Review on control techniques and methodologies for maximum power extraction from wind energy systems. *IET Renew Power Gener.* **12**, 1609–1622 (2018).

[3] Abdullah, M. A. et al. Towards green energy for smart cities: Particle swarm optimization based MPPT approach. *IEEE Access* **6**, 58427–58438 (2018).

[4] Hassan, M. et al. On-grid optimal MPPT for fine-tuned inverter-based PV system using golf optimizer considering partial shading effect. *Alexandria Eng. J.* **103**, 180–196 (2024).

[5] Mojallizadeh, M. R. & Badamchizadeh, M. A. Second-order fuzzy sliding-mode control of photovoltaic power generation systems. *Sol. Energy* **149**, 332–340 (2017).

[6] Lee, J. & Kim, Y.-S. Sensorless fuzzy-logic-based maximum power point tracking control for a small-scale wind power generation system with a switched-mode rectifier. *IET Renew. Power Gener.* **10**, 194–202 (2016).

[7] Hussain, J. & Mishra, M. K. Adaptive maximum power point tracking control algorithm for wind energy conversion systems. *IEEE Trans Energy Convers* **31**, 697–705 (2016).

[8] Lopez-Flores, D. R., Duran-Gomez, J. L. & Chacon-Murguia, M. I. A mechanical sensorless MPPT algorithm for a wind energy conversion system based on a modular multilayer perceptron and a processor-in-the-loop approach. *Elect. Power Syst. Res.* **186**, 106409 (2020).

[9] Chen, J. et al. Design of a unified power controller for variable speed fixed-pitch wind energy conversion system. *IEEE Trans. Ind. Electron* **63**, 4899–4908 (2016).

[10] Youssef, A.-R. et al. Advanced multi-sector P&O maximum power point tracking technique for wind energy conversion system. *Int. J. Electr. Power Energy Syst.* **107**, 89–97 (2019).

[11] Zribi, M., Alrifai, M. & Rayan, M. Sliding mode control of a variable- speed wind energy conversion system using a squirrel cage induction generator. *Energies* **10**, 604 (2017).

[12] Bayhan, S., Abu-Rub, H. & Ellabban, O. Sensorless model predictive control scheme of wind-driven doubly fed induction generator in dc microgrid. *IET Renew. Power Gener.* **10**, 514–521 (2016).

[13] Yaylacı, E. K. & Yazıcı, İ. Sensorless double integral sliding mode MPPT control for the WECS. *J. Renew. Sustain. Energy* **10**, 023301 (2018).

[14] Li, H. & Chen, Z. Overview of different wind generator systems and their comparisons. *IET Renew. Power Gener.* **2**, 123–138 (2008).

[15] Yazici, İ & Yaylaci, E. K. Maximum power point tracking for the permanent magnet synchronous generator-based WECS by using the discrete-time integral sliding mode controller with a chattering-free reaching law. *IET Power Electron* **10**, 1751–1758 (2017).

[16] Kumar, D. & Chatterjee, K. A review of conventional and advanced MPPT algorithms for wind energy systems. *Renew. Sustain Energy Rev.* **55**, 957–970 (2016).

[17] Liu, H., Locment, F. & Sechilariu, M. Experimental analysis of impact of maximum power point tracking methods on energy efficiency for small-scale wind energy conversion system. *IET Renew. Power Gener.* **11**, 389–397 (2017).

[18] Xia, Y., Ahmed, K. H. & Williams, B. W. Wind turbine power coefficient analysis of a new maximum power point tracking technique. *IEEE Trans. Ind. Electron* **60**, 1122–1132 (2013).

[19] Ganjefar, S., Ghassemi, A. A. & Ahmadi, M. M. Improving efficiency of two-type maximum power point tracking methods of tip-speed ratio and optimum torque in wind turbine system using a quantum neural network. *Energy (Oxf)* **67**, 444–453 (2014).

[20] Barakati, S. M., Kazerani, M. & Aplevich, J. D. Maximum power tracking control for a wind turbine system including a matrix converter. *IEEE Trans Energy Convers* **24**, 705–713 (2009).

[21] Sabzevari, S. et al. MPPT control of wind turbines by direct adaptive fuzzy-PI controller and using ANN-PSO wind speed estimator. *J Renew Sustain Energy* **9**, 013302 (2017).

[22] Lin, W.-M. & Hong, C.-M. Intelligent approach to maximum power point tracking control strategy for variable-speed wind turbine generation system. *Energy (Oxf)* **35**, 2440–2447 (2010).

[23] Wei, C. et al. An adaptive network-based reinforcement learning method for MPPT control of PMSG wind energy conversion systems. *IEEE Trans Power Electron* **31**, 7837–7848 (2016).

[24] El Aissaoui, H., El Ougli, A. & Tidhaf, B. MPPT using PSO technique comparing to fuzzy logic and P&O algorithms for wind energy conversion system. *Intellect. J. Energy Harvest. Storage.* **17**(1), 26–37 (2025).

[25] Yazıcı, İ & Yaylacı, E. K. Modified grey wolf optimizer based MPPT design and experimentally performance evaluations for wind energy systems. *Eng. Sci. Technol. Int. J.* **46**, 101520 (2023).

[26] Abolvafaei, M. & Ganjefar, S. Maximum power extraction from a wind turbine using second-order fast terminal sliding mode control. *Renew. Energy* **139**, 1437–1446 (2019).

[27] Dursun, E. H. & Kulaksiz, A. A. Second-order sliding mode voltage-regulator for improving MPPT efficiency of PMSG-based WECS. *Int. J Elect. Power Energy Syst.* **121**, 106149 (2020).

[28] Ahmed G, Abo-Khalil A, Sobhy K.Multiple Linear Regression Approach for Sensorless MPPT of PMSG Wind Power Generation Systems*, Res. Sq*. (2022).

[29] Soufyane, B. et al. Fully robust sensorless control of direct-drive PMSG wind turbine feeding a water pumping system. *IFAC-PapersOnLine.* **53**(2), 12797–12802 (2020).

[30] Zakzouk, N. E. Continuous input current buck DC/DC converter for small-size wind energy systems featuring current sensorless MPPT control. *Sci. Rep.* **14**(1), 380 (2024). 38172527 10.1038/s41598-023-50692-2 PMC10764938

[31] Bouchakour A, Zarour L, Bessous N, et al. MPPT algorithm based on metaheuristic techniques (PSO & GA) dedicated to improve wind energy water pumping system performance”. In: Rabehi A, Alwabli A, El-Abd M, et al. (eds) *Scientific report journal*. 2024. 10.1038/s41598-024-68584-4 PMC11297299 39095570

[32] Rashmi, G. & Mary, L. M. A novel MPPT design for a wind energy conversion system using grey wolf optimization. *Measur, Electron, Comput. Commun.* **64**(4), 798–806 (2023).

[33] Ersagun Kürşat Yaylacı, F. Modified golden section search based MPPT algorithm for the WECS * Eng. Sci. Technol. J.* **24**, 1123–1133 (2021).

[34] Fathy, A., Alharbi, A. G., Alshammari, S. & Hasanien, H. M. Archimedes optimization algorithm based maximum power point tracker for wind energy generation system. *Ain Shams Eng. J.* **13**(2), 101548 (2022).

[35] Fathy, A. & El-Baksawi, O. Grasshopper optimization algorithm for extracting maximum power from wind turbine installed in Al-Jouf region”. *J. Renew. Sustain. Energy* **11**, 033303 (2019).

[36] Yessef M, Bossoufi B, Taoussi M, et al. Improved hybrid control strategy of the doubly-fed induction generator under a real wind profile. In: *Lecture Notes in Networks and Systems*. Cham: Springer International Publishing, 2021, pp. 1279–1290.

[37] Agushaka, J. O. et al. Greater cane rat algorithm (GCRA): A nature-inspired metaheuristic for optimization problems. *Heliyon* **10**, e31629 (2024). 38845929 10.1016/j.heliyon.2024.e31629 PMC11154226

[38] Yang, B. et al. Robust sliding-mode control of wind energy conversion systems for optimal power extraction via nonlinear perturbation observers. *Appl Energy* **210**, 711–723 (2018).

[39] Fathabadi, H. Novel maximum electrical and mechanical power tracking controllers for wind energy conversion systems. *IEEE J Emerg Sel Top Power Electron* **5**, 1739–1745 (2017).

[40] Mustapha, O. A. et al. A study of scientific publications on the greater cane rat (Thryonomys swinderianus, Temminck 1827). *Animal Model Exp Med* **3**, 40–46 (2020). 32318658 10.1002/ame2.12103 PMC7167232

[41] *Britannica.com*, https://www.britannica.com/animal/cane-rat. (accessed April 3, 2025).

[42] Van Der Merwe, M. Discriminating between thryonomys swinderianus and thryonomys gregorianus. *Afr. Afr Zool* **42**, 165–171 (2007).

[43] Dhiman, G. et al. A novel algorithm for global optimization: Rat swarm optimizer. *J Ambient Intell Humaniz Comput* **12**, 8457–8482 (2021).

[44] Kaliappan K, Sekar R, Ramesh G, et al. Performance evaluation of P&O and PSO-based MPPT for wind energy conversion systems. In: *Lecture Notes in Networks and Systems*. Singapore: Springer Nature Singapore, 2023, pp. 899–910.

[45] Yazıcı, İ & Yaylacı, E. K. Modified grey wolf optimizer based MPPT design and experimentally performance evaluations for wind energy systems. *Eng Sci Technol Int J* **46**, 101520 (2023).

[46] Maroufi, O., Choucha, A. & Chaib, L. Hybrid fractional fuzzy PID design for MPPT-pitch control of wind turbine-based bat algorithm. *Electr Eng (Berl, Print)* **102**, 2149–2160 (2020).

[47] Kumar D, Chatterjee K. Artificial bee colony based MPPT algorithm for wind energy conversion system.* In: 2016 IEEE 6th International Conference on Power Systems (ICPS). IEEE, 2016, pp. 1–6*.

[48] Mokhtari, Y. & Rekioua, D. High performance of maximum power point tracking using ant colony algorithm in wind turbine. *Renew Energy* **126**, 1055–1063 (2018).

[49] Mohammed, H., Hany, M. & Hasanien, S. Enhanced whale optimization algorithm for maximum power point tracking of variable-speed wind generators. *J. Appl. Soft Comput.*10.1016/j.asoc.2019.105937 (2020).

